# Nickel-based nanomaterials: a comprehensive analysis of risk assessment, toxicity mechanisms, and future strategies for health risk prevention

**DOI:** 10.1186/s12951-025-03248-7

**Published:** 2025-03-14

**Authors:** Xiaoting Zhou, Jiaqi Liao, Zipeng Lei, Huiqin Yao, Le Zhao, Chun Yang, Yan Zu, Yuliang Zhao

**Affiliations:** 1 CAS Key Laboratory for Biomedical Effects of Nanomaterials and Nanosafety, Institute of High Energy Physics, Chinese Academy of Sciences, Beijing, 100049 China; 2 National Center for Nanoscience and Technology, Beijing, 100190 China; 3 College of Life Sciences and Chemistry, Hunan University of Technology, Zhuzhou, 412007 China; 4 College of Basic Medicine, Ningxia Medical University, Yinchuan, 750004 China; 5 Clinical College of the Third Medical Center of Chinese PLA General Hospital, The Fifth Clinical Medical College of Anhui Medical University, Hefei, 230032 Anhui China

**Keywords:** Nickel-based nanomaterials, Human health effects, Environmental exposure, Occupational exposure, Nanotoxicology

## Abstract

Nickel-based nanomaterials (NBNs) have seen a surge in usage across a variety of applications. However, the widespread use of NBNs has led to increased human exposure, raising questions about their associated health risks, both in the short and long term. Additionally, the spread of NBNs in the environment has attracted considerable attention, emerging as a vital focus for research and development. This review aims to provide an in-depth assessment of the current understanding of NBNs toxicity, the mechanisms underlying their toxicological effects, and the strategies for mitigating associated health risks. We begin by examining the physicochemical properties of NBNs, such as particle size, composition and surface functionalization, which are key determinants of their biological interactions and toxicity. Then, through an extensive analysis of in vitro and in vivo studies, we highlight the adverse effects of NBNs exposure, including the generation of reactive oxygen species (ROS), oxidative stress, inflammation, cytotoxicity, genotoxicity, and immunotoxicity. To address the potential health risks associated with NBNs, we propose future strategies for risk prevention, including the development of safer nanomaterial designs, implementation of stringent regulatory guidelines, and advancement of novel toxicity testing approaches.

**Graphical Abstract:**

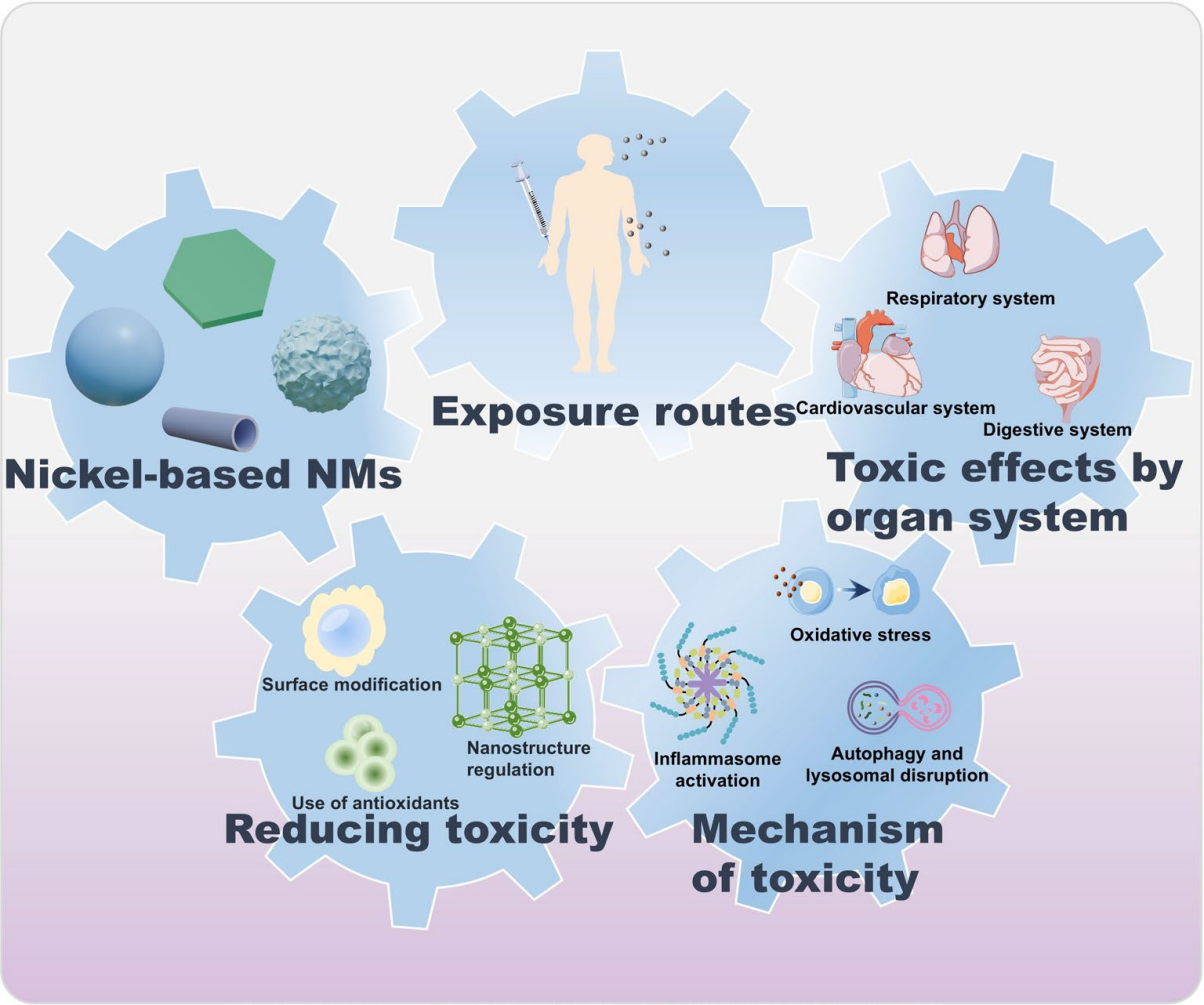

## Introduction

With the advancement of nanotechnology, nanomaterials (NMs) have found widespread applications in various fields including materials science, biomedicine, energy, environmental protection, electronics and optical devices. However, numerous nanomaterials have been found to pose risks to ecosystems and cause adverse health effects [1, 2]. Therefore, while nanotechnology offers numerous benefits, it is crucial to carefully assess and manage the potential risks to safeguard both the environment and public health.

Nickel-based nanomaterials (NBNs), renowned for their unique chemical and physical properties, demonstrate significant potential across a wide range of applications. Nickel nanoparticles (Ni NPs), in particular, are highly versatile. They function as efficient catalysts in chemical reactions, are integral to the production of conductive inks and electrode materials for electronic applications, and play a crucial role in biomedicine, where they are utilized for targeted drug delivery and magnetic resonance imaging [3, 4]. Nickel compound nanomaterials encompass a range of nanoscale materials formed by combining nickel with other elements. These primarily include nickel oxide nanoparticles (NiO NPs), nickel sulfide nanoparticles (such as NiS), and nickel ferrite (NiFe_2_O_4_), among others. Due to their unique electrochemical properties, catalytic performance, and magnetism, these materials hold significant value across various domains. These NBNs have a variety of commercial and research uses and the development of these nanomaterials has significantly advanced the fields of materials science, energy storage, and environmental management [5, 6]. Occupational exposure to nickel compounds in various workplaces can occur through skin contact or by inhaling aerosols, fumes, dust, or mists that contain nickel [7]. The toxicity of nickel compounds following experimental or industrial exposure, as well as the levels of daily nickel intake by humans, have been documented through various routes [8]. For example, a 26-year-old female worker experienced throat irritation, nasal congestion, post nasal drip, facial flushing while handling Ni NPs powder in a workplace that lacked specialized respiratory protection and control measures [9]. Research has shown that such occupational exposure to nickel compounds can lead to morphological changes in tissues. Because of the variety of manufacturing processes and waste streams, types and locations of use, types and amounts of wear during use, and methods of disposal, there is concern that the widespread use of NBNs poses certain environmental and health risks, particularly as they may be released into the environment and lead to human exposure.

Numerous studies have been conducted to assess the toxic effects and mechanisms of toxicity of NBNs [10]. Research on the toxicity of NBNs has identified significant health concerns, especially in occupational settings with higher exposure risks. In vitro and in vivo studies show that NBNs can cause cytotoxicity, oxidative stress, genotoxicity, and inflammation. Smaller particles tend to be more toxic due to their increased surface area and reactivity [11, 12]. Inhalation studies in animal models have shown respiratory and cardiovascular effects, while limited human data highlight symptoms such as respiratory irritation and skin sensitization among exposed workers. The toxicological profile of typical nanomaterials containing nickel (e.g. Ni and NiO NPs), has been extensively investigated [13, 14]. Reports from human and animal studies have indicated respiratory toxicity, carcinogenic potential, skin sensitization, and reproductive effects upon exposure to Ni and NiO NPs. The bioavailability of Ni^2+^ ions at target sites is considered a key factor influencing the varying toxicity levels observed among different chemical forms of NBNs.

**Fig. 1:**
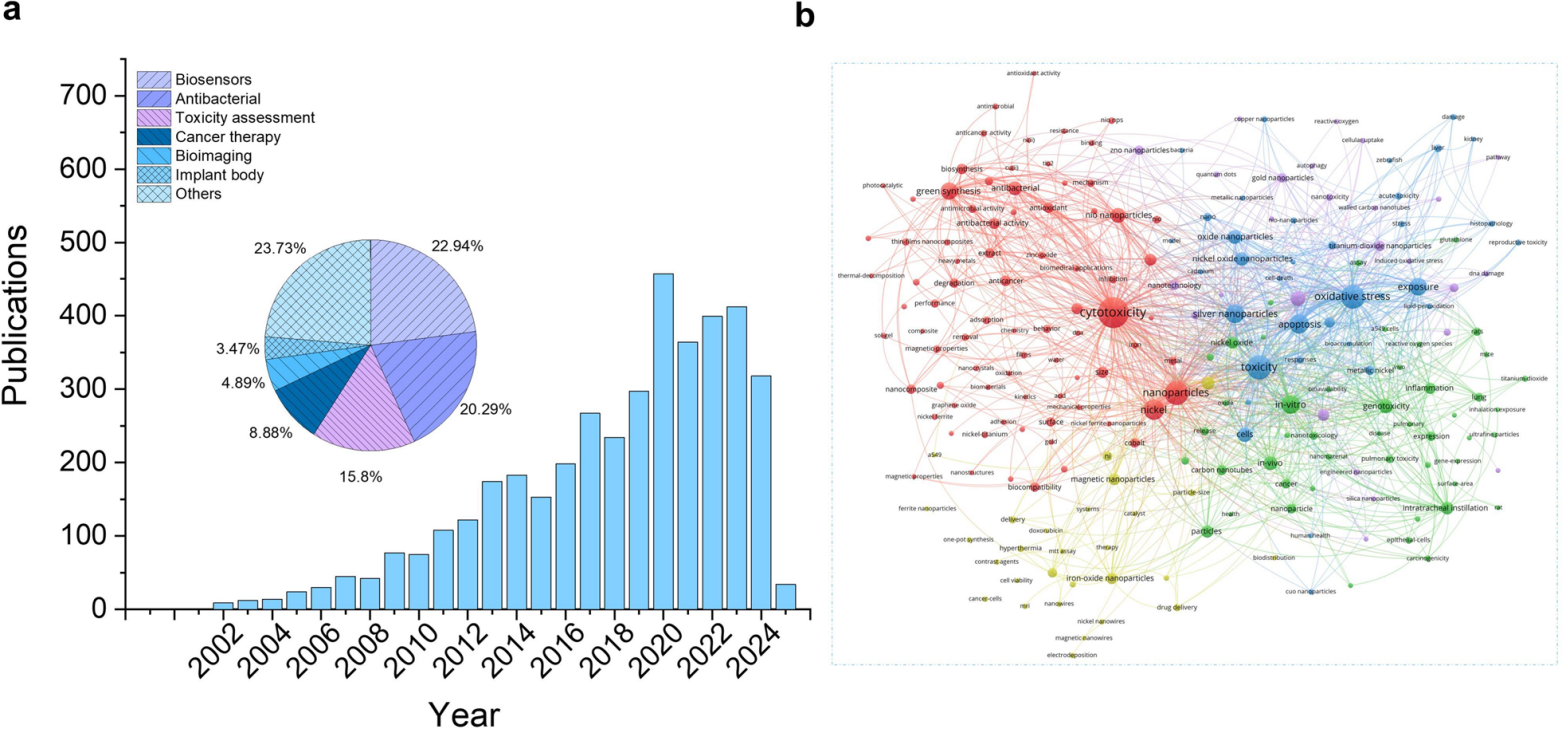
**a** Published papers on the interactions between NBNs and living organisms, organized by period, up to mid-January 2025. This embedded pie graph shows the proportions of different types of interactions between NBNs and living organisms. **b** The overlay visualization of co-occurrence analysis of the toxicity assessment of NBNs based on all keywords. Data is collected from the web of science. Node size suggests the document amount

Currently, many new NBNs have emerged, showing potential applications in various fields (Fig. 1). Therefore, understanding their biological toxicity is essential to provide references for their safety design. However, existing review articles either focus on the toxicity of Ni or NiO nanomaterials or are concentrated on a specific exposure route [10–13]. They do not offer a comprehensive understanding of the toxicity of NBNs on the environment or living organisms. Hence, this review will systematically introduce the physicochemical properties of NBNs, factors influencing their toxicity, exposure routes, toxicokinetics and toxicodynamics, and protective strategies. This review aims to provide references for the safety design of NBNs and the prediction of the toxicity of new NBNs.

## Physicochemical properties of NBNs

**Fig. 2:**
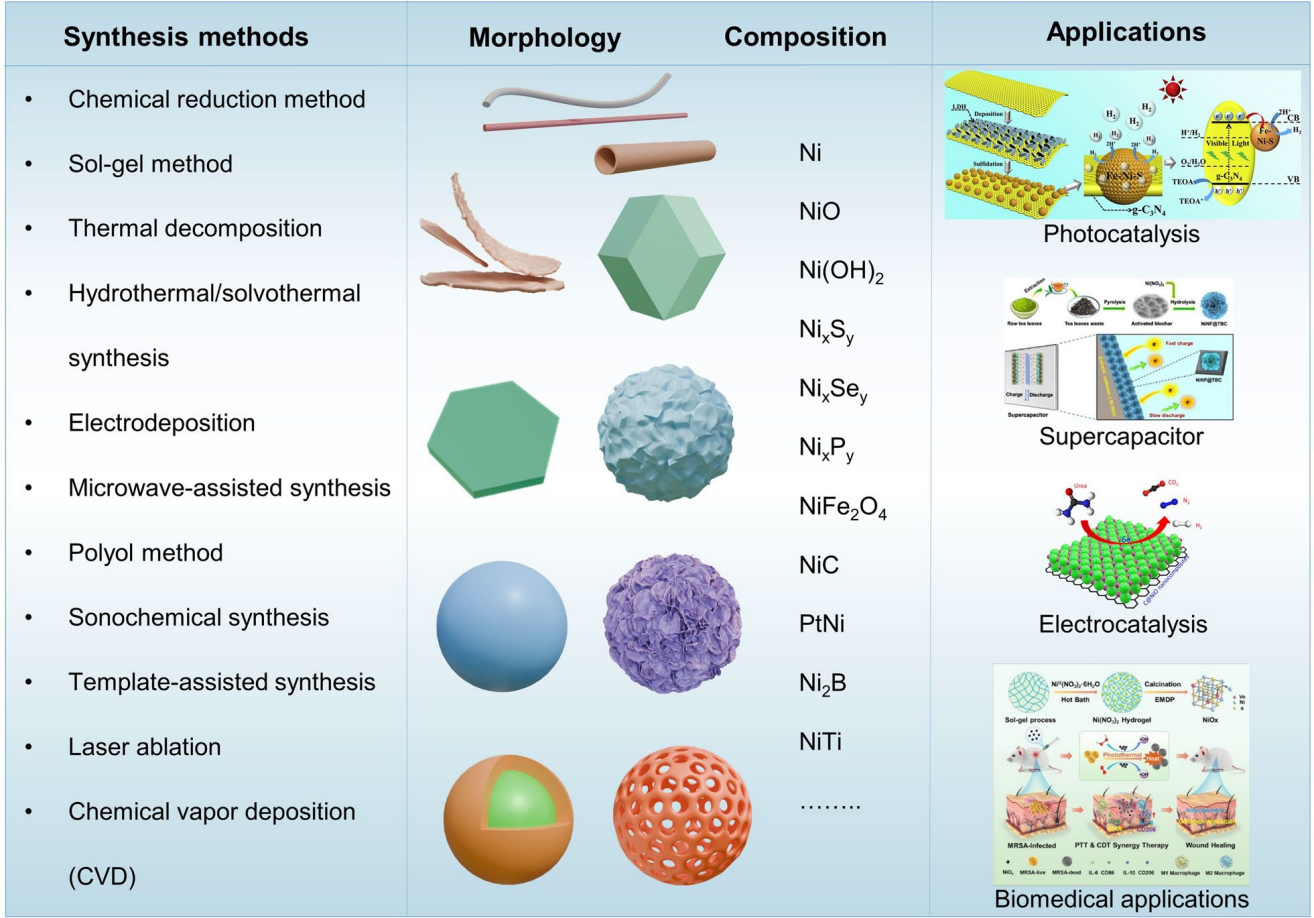
The wide-ranging applications of diverse NBNs [25–28]

The physicochemical properties collectively determine the performance and applications of NBNs, while also influencing their behavior and potential toxicity in environmental and biological systems. The main types of NBNs include Ni NPs, NiO NPs, nickel ferrite NPs (NiFe_2_O_4_), nickel sulfide NPs (NiS, NiS_2_, etc.), nickel–cobalt NPs, nickel phosphide NPs (Ni_2_P, Ni_12_P_5_, etc.), nickel selenide (NiSe, NiSe_2_, Ni_3_Se_2_, etc.). Each type of NBNs offers distinct properties and advantages, making them suitable for specific applications. Their synthesis, characterization, and application continue to be active areas of research in nanotechnology and materials science (Fig. 2). This section offers an overview of the physicochemical properties of NBNs, such as size, chemical composition and surface coating.

NBNs possess a wide range of physicochemical properties and can be synthesized in various shapes, such as nanospheres, nanorods, nanosheets and nanoflowers [15], each influencing their catalytic properties. In addition to the aforementioned main components, NBNs can also be enhanced by doping with other elements or by forming composites with other nanomaterials to achieve superior performance. Examples of these novel composite materials include NiSe_2_/CeO_2_, Fe_0.2_NiCo_1.8_Se_4_, Sn-doped Ni_3_S_2_ [16–18]. Surface modification of NBNs is a crucial strategy for enhancing their performance in various applications, including catalysis, sensing, energy storage, and biomedical applications. Some common surface modification techniques and objectives for NBNs include coating with protective layers** (**oxide coatings [19], polymer coatings [20–22], etc.**),** creating core–shell structures [23], etc. For example, the surface modification with polyaniline (PANI)-Ru@Ni_3_S_2_ exhibited catalytic hydrogen production activity similar to Ru@Ni_3_S_2_, but with remarkably improved stability [24]. In summary, NBNs possess a range of unique physical and chemical properties that make them attractive for a wide variety of applications. The ability to tailor these properties by controlling size, shape, composition, and surface modification not only expands their applications but also impacts their environmental and health effects.

## Factors influencing toxicity

The factors affecting the toxicity of nanomaterials are size, shape, chemical composition, surface properties, exposure time and concentration [29, 30], biological system factors, processing and treatment methods [31], etc. We have summarized primary particle size, size in media and morphology of representative NBNs used in toxicity studies (Table 1). The following mainly introduces some factors affecting the toxicity of NBNs.

**Table 1:** Primary particle size, size in media and morphology of representative NBNs used in toxicity studies

Nanoparticle type	Primary particle size (average ± SD)	Primary particle size in media (average ± SD)	Morphology	Refs.
NiO	Green 100 nm; Black 20 nm	Green (NR); black 38–180 nm	Spherical	[32]
NiO	50 nm	342 nm	Cubic	[33]
NiO	20 ± 5 nm	100–800 nm	Spherical	[34]
NiO	20–50 nm	350 nm	Spherical and cubical	[35]
NiO	50–100 nm	323.8 ± 56.67 nm	Oval and cubical	[36]
NiO	20 nm	244.5 nm	Spherical	[37]
Sr-NiO	21 nm	347.94 nm	Spherical and cubic	[38]
NiO	21.6 ± 3.6 nm 30 ± 1.4 nm	346 ± 3.2 nm 197 ± 2.8 nm	Cubic	[30]
Ni	10–30 nm	250 nm	Spherical	[39]
C-coated Ni	10–30 nm	274.8 nm	Core–shell	[40]
Ni	73.94 ± 2.61 nm	86.75 ± 5.26 nm 841.3 ± 16.76 nm	Spherical	[41]
Ni	30–100 nm	260–725 nm	Spherical	[42]
Ni	20–150 nm	90–615 nm	Spherical	[43]
NiTi	64 nm	166.4 nm	Spherical	[44]
Ni	25.43 ± 11.62 nm	250–600 nm	Spherical	[45]
NiPt	< 50 nm	45.1 ± 1.2 nm	Dodecahedron	[46]
Ni	22.3 ± 8.0 nm	170.0 nm	Spherical	[47]
Ni	20 nm	250 nm	Spherical	[48]
Ni	55.8 ± 14.0 nm	181.6 ± 4.6 nm	Spherical	[49]
FeNi	42.26 ± 17.70 nm	180 nm	Spherical	[50]
NiFe_2_O_4_	31.2 nm	NR	Spherical	[51]
Ni_2_B	Micro-sized to nanosized	NR	Spherical and irregular cubic	[52]
NiSe_2_/rGO	200 nm	957.7 ± 28.5 926.8 ± 20.4	Hollow nanosphere	[53]
Ni(OH)_2_	100 ± 20 nm	NR	Nano-chips shaped	[54]

### Size

Size, structure, and morphology are physicochemical properties that significantly influence the biological effects of nanomaterials. The size of nanomaterials plays a crucial role in determining their toxicological properties. Smaller NPs generally exhibit higher toxicity due to their increased surface area, enhanced cellular uptake, and greater potential for inducing oxidative stress and other harmful biological effects [55]. Compared to microparticles, NBNs also exhibit greater toxic effects [56–58]. Singh et al. investigated the effects of different sizes of NiO NPs on the kidneys. Their results indicated that NiO NPs induce stronger nephrotoxic effects compared to micron-sized NiO, with oxidative stress playing a major role in this process. ROS are the key factors leading to NiO NPs-induced nephrotoxicity in rats [59]. The study by Nishi et al. suggested that NiO NPs exhibited greater pulmonary toxicity compared to micron-sized NiO, likely because they dissolve more slowly, releasing more Ni^2+^ ions [60]. Specifically, Abdulqadir et al. meticulously examined the anticipated adverse effects of Ni NPs of various sizes on the renal units of rat kidneys [61]. The experimental animals were intraperitoneally exposed to Ni NPs of three different sizes (20 nm, 40 nm, and 70 nm) on a daily basis. Based on the findings of this in vivo study, it was concluded that rats injected intraperitoneally with Ni NPs exhibited significant oxidative stress, lipid peroxidation, inflammation, and renal tubular cell degeneration. These nephrotoxic effects were size-dependent, with the smallest NPs (20 nm) demonstrating more pronounced nephrotoxic effects compared to the larger sizes (40 nm and 70 nm).

In addition to the aforementioned studies on the toxicity of spherical NBNs of varying sizes, research has also been conducted on the toxicity of fibrous NBNs with different lengths. Poland et al. confirmed the length-dependent pathogenicity of fibrous nickel nanowires [62]. Their findings indicated that synthesized nickel nanowires, predominantly long (> 20 microns), could induce strong dose-dependent inflammation in a mouse peritoneal model. In contrast, no inflammation or fibrosis was observed with short nanowires (< 5 microns). This length-dependent response was also evident following lung aspiration and in an in vitro macrophage model, further demonstrating that fiber length is a crucial determinant of potential hazard. These findings are significant for assessing the risks posed by fibrous nanomaterials and for their regulation in occupational settings.

### Composition

Changes in the composition of nanomaterials can significantly impact their toxicity by altering their chemical reactivity and biological interactions [63]. There have also been some reports on studying the toxicity of NBNs through changes in their composition. In a study, Faisal and colleagues utilized Curcuma longa extract to synthesize a diverse array of NPs, encompassing CuO, NiO, and Cu/Ni hybrids [64]. These NPs underwent rigorous assessment across a spectrum of biological applications, including combatting urinary tract infection (UTI) isolates, showcasing anti-leishmanial effects, exploring anti-diabetic properties, evaluating antioxidant capabilities, investigating anti-cancer activities, and gauging biocompatibility. The findings unveiled the robust efficacy of CuO, NiO, and their hybrid counterparts against multidrug-resistant UTI isolates, surpassing the performance of traditional antibiotics. Notably, the hybrid NPs, in particular, exhibited significant anti-leishmanial activity and cytotoxicity against the human liver cell line (HepG2). Specifically, the Cu/Ni hybrids demonstrated a superior inhibition rate of 73.18 ± 2.42% against fresh HepG2 cell lines, in contrast to 64.10 ± 1.91% for CuO NPs and 47.55 ± 1.61% for NiO NPs. The cytotoxic effects of these NPs stem from three primary mechanisms: their dissolution into functional entities, the induction of ROS generation, and DNA damage.

In the context of novel medical and drug delivery applications, Alsamhary used a green method to prepare pure NiO and 5% cobalt-doped NiO (Co-NiO) NPs [65]. They observed that the particle size of the Co-NiO NPs was approximately 80 nm. The cytotoxicity assessment revealed that Co-NiO NPs exerted a more pronounced effect on breast cancer cells (MCF-7) compared to NiO NPs. This indicates that the doping process of NiO NPs leads to an increased inhibitory effect on MCF-7 cells. Similarly, Hamidian and colleagues proposed a green method for synthesizing pure and 1%, 3%, and 5% cobalt-doped NiO nanoparticles (Co-NiO NPs) [66]. Their findings indicated that upon doping NiO structures with cobalt ions, the absorption peaks of NiO NPs exhibited a blue shift. Based on toxicity tests conducted on the synthesized NPs against MCF-7 and human umbilical vein endothelial (HUVEC) cells, the Co-doped NPs demonstrated higher cytotoxic activity compared to the pure NPs.

### Surface modification

Surface chemistry plays a pivotal role in the agglomeration state of NPs, with the adsorption of proteins being a key factor that can modify biological interactions. The preparatory treatment of NPs before toxicological evaluation is also known to impact study results [67]. Different research laboratories have implemented varying dispersion methods to investigate the biological response to NBNs in vitro and in vivo. Most studies have employed dispersion media to ensure NPs are well-dispersed for in vivo experiments. In a study, the short-term exposure to Ni NPs may result in acute lung inflammation and injury, whereas prolonged exposure could lead to chronic lung inflammation and fibrosis. The application of surface modifications to Ni NPs, particularly carbon coatings, has been shown to mitigate the pulmonary effects induced by these NPs, significantly reducing both acute and chronic lung inflammation and injury [39]. In addition, Mo et al. demonstrated that wild-type mouse primary monocytes exposed to 30 µg/mL Ni NPs for 24 h exhibited a significant increase in MMP-2 and MMP-9 production. They observed that Ni NPs and nano-nickel-p caused upregulation of microRNA-21(miR-21) in WT monocytes, whereas nano-nickel-c did not trigger these effects. This indicated that surface modifications, such as carbon coatings on Ni NPs, can attenuate nickel-induced upregulation of miR-21 and matrix metalloproteinases [40]. Copolymer coating such can inhibit the release of Ni^2+^ ions from NiTi alloy so that it can improve the safety and success rate of NiTi-based implantations. By modifying the surface of Ni NPs with a gold shell, the gold shell can protect the Ni NPs core from oxidation, thereby reducing its toxicity.

Moreover, Cheng and colleagues constructed nickel nanotubes with varying thicknesses of silica nanoshells [68]. They investigated the effects of silica layer thickness, incubation time, and cell line type on the cytotoxicity of the synthesized materials and evaluated their biocompatibility using bioenzymes. In their toxicity experiments, different cell lines containing both tumor and normal cells were used, confirming the low cytotoxicity and good biocompatibility of Ni@SiO_2_. To achieve efficient immobilization and purification of histidine-rich proteins, Ni@SiO_2_-NH_2_ was obtained by introducing amino functional groups. Compared to other synthesized materials, Ni@SiO_2_-NH_2_ exhibited lower cytotoxicity and higher adsorption capacity. This work not only provides insights into reducing the cytotoxicity of bionanomaterials and improving their biocompatibility but also lays the foundation for subsequent biological applications.

### Dissolution

The release of toxic metal ions from metal nanomaterials is a significant factor influencing their toxicity [69]. This ion release can lead to increased cellular damage and oxidative stress, contributing to the overall harmful effects of the nanomaterials. NiO NPs, as typical metal oxide NPs, have been shown to exhibit greater lung toxicity than their micron-sized NiO counterparts due to the slow dissolution rate that produces a higher yield of Ni^2+^ ions. This was confirmed by Nishi et al. [60] who revisited previous intratracheal instillation studies in rats, focusing on nickel retention in the lungs and the lung-to-body weight ratio, alongside measuring the solubility of NiO NPs and micron-sized NiO in artificial lysosomal fluid (ALF) at pH 4.5. The dissolution of NiO NPs in ALF occurs over approximately one week, whereas in the body, this process extends to a month or longer. This leads to the conclusion that the slow dissolution of NiO NPs within the phagolysosomes of alveolar macrophages results in the production of Ni^2+^ ions. These ions cause the macrophages to become foam cells after one month, with the inflammatory response persisting for up to three months post-instillation. This highlights the critical role of dissolution kinetics in NiO NP-induced lung toxicity.

In the anti-tumor application of NBNs, the production of dissolved and released Ni^2+^ ions can also play an important role in killing tumor cells. A multifunctional nanoplatform was engineered utilizing mesoporous NiO (mNiO) NPs and terbium complexes, serving as a carrier for artemisinin (ART), a T2-weighted contrast agent, and a luminescent imaging probe [70]. The unique pH-responsive nature of mNiO enables it to degrade and release Ni^2+^ ions specifically in the acidic tumor microenvironment (TME). The inherent peroxide bridge bonds present in the structure of ART have a propensity to interact with Ni^2+^ ions, leading to the generation of free radicals that possess the capability to induce cell death in tumor cells. It is important to note that nanomaterials with higher solubility may more readily decompose in the body and release toxic substances, while more persistent nanomaterials may accumulate in the body, leading to long-term toxic effects.

### Concentrations, doses, and time

The concentration or dose of nanomaterials significantly affects their toxicity [71]. In the toxicity assessment of NBNs, both at the cellular and in vivo levels, toxicity generally increases with the concentration of the nanomaterials [72, 73]. For example, Ahmad et al. investigated the cytotoxicity of Ni NPs using the HepG2 cells and found that Ni NPs, at concentrations ranging from 25 to 100 µg/mL, could induce oxidative stress and apoptosis in a dose-dependent manner. In another study, human lung epithelial cells (A549) cells treated with Ni NPs exhibited decreased cell viability. At the same dose, Ni NPs demonstrated greater genotoxicity compared to nickel fine particles (Ni FPs). Additionally, Ni NPs were more effective than Ni FPs in activating oncogenes and causing dose-dependent DNA damage [71]. The toxicity of NBNs generally increases with prolonged exposure time. Moreover, Iftikhar and colleagues assessed the male reproductive toxicity of Ni NPs in Sprague Dawley rats [74]. Their study used spherical Ni NPs with an average particle size of 56 nm. Twenty-five healthy rats (weighing 200–220 g) were divided into a negative control group (0), a placebo group (0.9% saline), and three treatment groups (15, 30, and 45 mg per kg of body weight). Their results showed that higher doses (45 mg/kg body weight) of Ni NPs significantly reduced body weight, serum testosterone levels, and daily sperm production. Meanwhile, the testicular index, nickel accumulation, and histological changes in testicular tissue (including basement membrane and seminiferous tubule necrosis, and vacuole formation) increased with the dose of Ni NPs.

Exposure duration is also typically an important factor influencing the toxicity of NBNs [49, 56, 59]. For example, Liu and colleagues verified the cytotoxicity of NiSe_2_/rGO nanocomposites with different surface defects under more realistic exposure conditions [53]. Compared to short-term and repeated exposures, long-term exposure to NiSe_2_/rGO led to enhanced oxidative stress, mitochondrial dysfunction, DNA damage, and calcium homeostasis disruption in rat lung macrophages. Notably, no significant differences were found between NiSe_2_/rGO with different surface defects, indicating that the type of defects in nanomaterials is not an accurate predictor for realistic risk assessment. Overall, this study provides insights into the true potential toxic effects and exposure thresholds of nanomaterials and calls for new perspectives in the risk assessment of engineered nanomaterials under long-term exposure, which differs from traditional short-term and repeated exposure assessments.

### Environmental factors

Engineered nanomaterials, can be influenced by a variety of environmental factors such as pH, ionic strength, presence of natural organic matter, temperature, light exposure, presence of other contaminants or chemicals, biological interactions etc. [75]. These factors can alter the behavior, bioavailability, and ultimately the toxicity of these nanomaterials in the environment. The biological toxicity effects of NBNs are also influenced by these external environmental factors. At present, environmental pollutants are indeed a very important problem. Environmental pollutants can have a serious impact on the ecosystem and human health. There are also some studies on co-exposure influence of NBNs and environmental pollutants on life [76, 77]. For example, Benzo[a]pyrene (BaP) is a widespread pollutant commonly found in cigarette smoke, diesel exhaust, and grilled foods, which are the primary sources of BaP exposure. Ahamed and colleagues investigated the effects of co-exposure to NiO NPs and BaP in HepG2 cells and primary rat hepatocytes [78]. Their study revealed that both individual and combined exposures to NiO NPs and BaP led to cytotoxicity, lactate dehydrogenase leakage, lipid peroxidation, depletion of mitochondrial membrane potential, and activation of caspases (−3 and −9) in both cell types. Furthermore, these exposures accelerated the production of free oxygen radicals and the depletion of antioxidants such as glutathione (GSH) and various antioxidant enzymes. Notably, the combination of NiO NPs and BaP exhibited synergistic toxicity in HepG2 cells and primary rat hepatocytes. This combined toxicity was primarily mediated through oxidative stress induced by ROS. These findings underscore the need for further research into the risk assessment of co-exposure to NiO NPs and BaP using suitable in vivo models.

For NBNs, an external magnetic field is another important factor influencing their biological toxicity. Magnetic patterning and responsive biomaterials have exhibited the capability to induce localized cell death in response to magnetic signals in a controlled manner [79]. These innovative nanostructured biomaterials feature magnetic nanoparticle-vesicle assemblies (MNPVs), which are composed of thermosensitive vesicles crosslinked by magnetic NPs. Acting as nanoscale drug delivery platforms, MNPVs can release encapsulated chemical messengers upon exposure to an alternating magnetic field (AMF), enabling precise and targeted therapeutic interventions. The magnetically triggered release of Ni^2+^ ions from MNPVs embedded in alginate hydrogels has been utilized to remotely trigger and spatially control apoptosis in fibroblasts cultured within the hydrogel.

Furthermore, external light sources can also enhance the cytotoxic effects of NBNs on tumors. Qian et al. described a magneto-solvothermal approach to adjust the crystallinity and morphology of polyethylene glycol (PEG)-ylated urchin-like nickel nanoclusters (referred to as 9T-PUNNC) under a high magnetic field with an intensity of 9T, aimed at enhancing combined photothermal-chemodynamic therapy (CDT) [80]. Their research findings demonstrated that the needle-like protrusions on the surface of 9T-PUNNC significantly increased the absorption of near-infrared (NIR) light in the NIR-II window and converted it into localized hyperthermia, achieving effective photothermal therapy (PTT). The light and heat produced by NIR-II further facilitated the release of Ni^2+^ ions, thereby enhancing the efficacy of Ni^2+^-mediated CDT. Both in vitro and in vivo results indicated that, under NIR-II irradiation, the combined therapy’s synergistic effects enabled 9T-PUNNC to effectively kill tumor cells and inhibit tumor growth.

Understanding the influence of these environmental factors on the toxicity of NBNs is essential for developing safer nanotechnologies and for conducting accurate risk assessments. It highlights the complexity of nanomaterials' behavior in the environment and the need for comprehensive, context-specific studies to evaluate their potential impacts. We will discuss in detail later in this article how active substances in the environment can mitigate the toxicity of NBNs.

## Exposure routes of NBNs

**Fig. 3:**
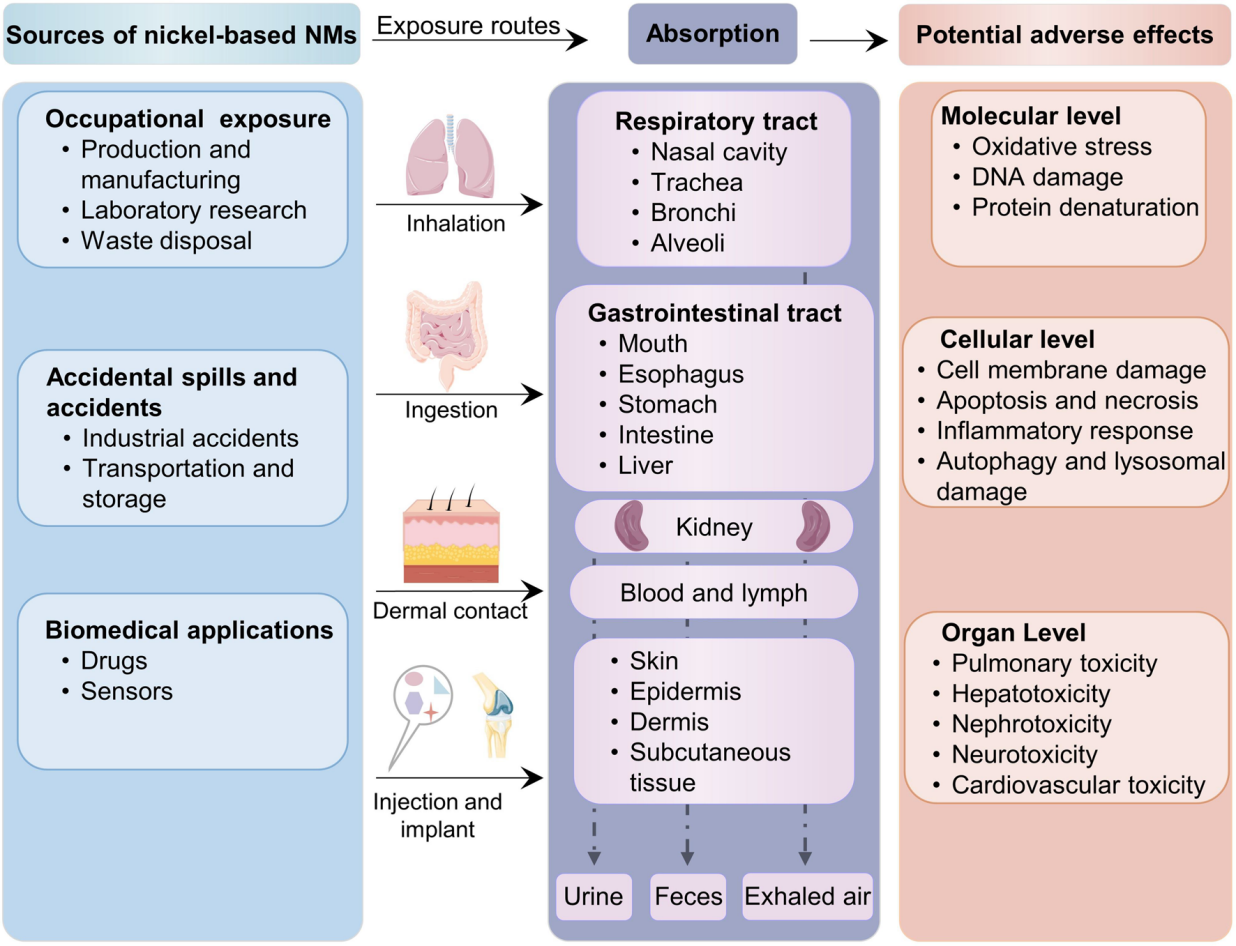
Sources of risk, exposure pathways, and potential hazards of NBNs

Nanomaterials containing nickel can pose exposure risks in various settings, particularly in industries and applications where they are manufactured, handled, or utilized. The main exposure sites for these nanomaterials include occupational settings, industrial applications, and waste management [81, 82]. Nanomaterials can enter the human body and the environment and produce negative effects through several exposure pathways (Fig. 3). The main exposure pathways for nanomaterials are introduced as follows.

### Inhalation

Inhalation stands as the most potent pathway for entry, with NPs deftly bypassing mucociliary defenses to settle in the depths of the alveolar region. Yet, the influence of inhaled NPs extends well beyond the pulmonary boundaries, manifesting notably in highly vascularized organs like the cardiovascular system and the brain, where their effects become particularly pronounced [83]. There is limited data on human and worker exposure to Ni NPs, but a recent case reported that a worker developed occupational asthma after handling nanoscale nickel particles without appropriate protective measures, as recommended for workers exposed to NPs [9]. Studies have reported a case where a man died after inhaling Ni NPs, with the cause of death being adult respiratory distress syndrome (ARDS). Ni NPs smaller than 25 nm were found in macrophages in his lungs, indicating significant uptake and accumulation of NBNs in the lungs [84]. This indicates that NBNs can be significantly taken up and accumulated in the lungs.

Ni and NiO NPs have been the focus of extensive research [85], particularly concerning their impact on the respiratory system. Various assessment models, including human lung epithelial cells (BEAS-2B) [86, 87], lung cancer cells (A549) [38, 88], and live subjects such as rats and mice, have been employed to investigate the consequences of exposure via inhalation. The range of potential injuries identified includes cellular processes like apoptosis and ferroptosis, as well as acute and chronic pulmonary inflammation, injury, and fibrosis. Additionally, the genotoxic and carcinogenic effects of these NPs have been highlighted, underscoring the need for careful consideration of their use and the importance of protective measures in environments where exposure is possible.

There have also been many reports on in vivo studies of respiratory exposure to NBNs. For instance, literature has detailed the toxicological mechanisms that may occur within the body upon inhalation of Ni NPs. These NPs have been pinpointed as triggers for DNA damage. When coupled with existing deficiencies in DNA repair processes via the hypoxia-inducible factor 1α (HIF-1α)/miR-210/RAD52 pathway, this can lead to the onset of genomic instability [73]. This sequence of molecular interactions may ultimately result in cellular transformation, underscoring the complex and potentially hazardous effects of Ni NPs at the cellular level.

### Ingestion

The risks associated with the unintentional intake of nanomaterials are further exacerbated by inadequate labeling or improper packaging, which can mislead and expose consumers to potential hazards [89, 90]. Persistent ingestion of nanomaterials raises alarms for health, as emerging studies indicate possible detrimental effects on critical systems such as the immune, neurological, and respiratory frameworks. Notably, the environmental presence of NBNs and the health implications of their consumption have garnered scientific scrutiny. For example, in dentistry, nickel is found in orthodontic appliances for moving teeth, stainless steel for tooth reinforcement, and clasps. When these materials are applied in the oral cavity and rotary tools are used for grinding, Ni NPs may be generated, potentially posing health risks to the body [47]. Additionally, Ni NPs are used in the food industry as a catalyst in the hydrogenation of fats, so the potential residue of nickel-containing catalysts in food prompts concerns about the oral exposure risks of NBNs [91].

By using oral administration techniques to evaluate the toxicity of nanomaterials, we can more accurately simulate the exposure scenarios in real-world applications, thus providing toxicity data that is more physiologically relevant. In a study by Kong and colleagues, male and female Sprague–Dawley (SD) rats were orally administered Ni NPs via gavage at doses of 5, 15, and 45 mg/kg/day. The researchers documented significant ultrastructural changes in the ovaries and testicles of the rats, accompanied by alterations in markers related to oxidative stress and aberrant expression of proteins linked to apoptosis. Furthermore, the study highlighted the antioxidant capabilities of vitamin C. Notably, the investigation revealed that Ni NPs impacted the motility and velocity of sperm in SD rats and led to a decrease in sex hormone levels.

### Skin

Nanomaterials, with their minuscule size, have the unique ability to navigate past the skin’s natural defenses, prompting a diverse array of biological reactions [92]. These can manifest as dermatological irritations, hypersensitivity, or even direct cytotoxicity, affecting the skin’s integrity and health. Certain nanomaterials are known to provoke reactions such as inflammation and itchiness, while others may have deeper, more insidious effects on the viability and functionality of skin cells.

Nickel-induced contact dermatitis is a severe allergic reaction to objects or environments that contain nickel. NBNs, with their broad industrial applications in the realms of energy storage, catalysis, and sensing technologies, raise particular concerns regarding dermal exposure. Throughout the lifecycle of these materials, from production to disposal, they may come into direct contact with human skin, prompting significant scientific investigation into the implications of such interactions. Data on human and worker exposure to Ni NPs are limited, but a recent case highlighted that a worker developed contact dermatitis after handling Ni NPs without following recommended protective measures for nanoparticle exposure. The worker managed nanoscale nickel without utilizing a local exhaust ventilation system or a glove box, and she did not wear a respiratory mask intended to safeguard against NPs exposure [9, 93]. The skin absorption of Ni NPs is higher compared to bulk nickel. It has been reported that a worker experienced an allergic reaction while handling Ni NPs [94].

Research indicates that epidermal keratinocyte exposure to Ni NPs can result in the release of Ni^2+^ ions [95], accumulating at the cell membrane and associated with the synthesis of a nickel-chelating peptide, implicated in the modulation of genes like the tumor-associated P63 regulatory gene 1 protein, at cytotoxic concentrations. A comprehensive, nuanced research strategy is imperative to unravel the complexities of dermal exposure to NBNs.

### Intravenous injection

The benefits of intravenous injection of nanomaterials include enhanced drug targeting and efficacy, reduced side effects, improved bioavailability and stability of drugs, and increased accuracy in medical imaging and diagnostics [96]. These advantages make nanomaterials highly promising for precision medicine and advanced therapeutic applications. The direct injection of NBNs into the bloodstream is typically associated with the administration of multifunctional NBNs for medical purposes. Medical-grade NBNs undergo rigorous evaluation and testing prior to clinical application and are often engineered with inherent properties and surface modifications to minimize the likelihood of adverse effects. In a study by Hu and colleagues, a novel NIR-II response nanoplatform, specifically nickel selenide@polydopamine nanocomposites (NiSe@PDA NCs), was developed through the in-situ coating of polydopamine (PDA) onto the surface of biomimetic NiSe NPs for dual-mode image-guided PTT [97]. Tumor-bearing mice received intravenous administration of the NiSe@PDA NCs in the study. The time-dependent magnetic resonance imaging (MRI) and thermal imaging were used to evaluate the accumulation behavior of NiSe@PDA NCs. In this study, the authors demonstrated that the nanoagents were primarily cleared by the reticuloendothelial system and excreted in the stool. During the whole treatment period, NiSe@PDA nanocrystals showed good biocompatibility and no obvious toxicity.

Liu and collaborators designed a nanoplatform utilizing mNiO NPs and mNiO terbium complexes (mNiO–Tb) for the delivery of ART in cancer theranostics [70]. The pH-responsive nature of mNiO allowed for the degradation and release of Ni^2+^ ions specifically in the acidic TME. The interaction between the peroxide bridge bonds in ART and Ni^2+^ ions led to the generation of free radicals with the potential to eradicate tumor cells. Additionally, owing to its remarkable NIR absorbance, mNiO was explored as a promising photothermal conversion agent for cancer PTT. The in vivo toxicity and efficacy of mNiO–Tb were assessed, demonstrating no deaths or observable abnormal behavior even at high doses. Serum biochemical tests were conducted on mice at various time points post-injection of mNiO–Tb revealed normal liver and kidney function indexes, indicating no biological dysfunction. These results suggest that mNiO–Tb holds promise as a low-toxicity and biocompatible therapeutic tool for cancer treatment.

In addition to the above PTT, NBNs are also used in photodynamic therapy (PDT) through intravenous injection. In order to solve the problem of high systemic toxicity and low selectivity in the treatment of gastric cancer, Wang group constructed an intelligent multifunctional nanoplatform (NNPIP NPs) with synergistic effects of PTT and PDT, which is composed of nickel/nickel phosphide (Ni/Ni–P) nanospheres as the core, polyethyleneimine (PEI) as the shell and loaded with the photosensitizer indocyanine green (ICG). Co-delivery of ICG and NPs into cells enhances PDT effect by reducing singlet oxygen (^1^O_2_) consumption. Finally, the treatment strategy of injecting NNPIP NPs into the body by intravenous injection not only reduced the tumor, but even completely eliminated the tumor in a quarter of the samples. In fact, as a carrier, the NBNs here can still be loaded with other plant-derived photosensitizers with good safety and easy degradation in the future, such as plant-derived photosensitizers (hypocrellins, carotenoids, curcumin, etc.) [98], thus improving the safety of NBNs through intravenous injection.

The exposure risks associated with intravenous injection of nanomaterials primarily include acute and long-term toxic reactions, immune system hypersensitivity or suppression, organ accumulation and damage, cytotoxicity, blood compatibility issues (such as coagulation disorders and hemolysis), and potential drug interactions. These risks need to be managed through comprehensive research and stringent safety regulations to ensure their safety in medical applications.

## Toxicokinetics and toxicodynamics

### Absorption, distribution, and uptake

By studying how nanomaterials are absorbed, distributed, and taken up by biological systems, researchers can gain insights into their interactions with cells, tissues, and organs [99, 100], This knowledge is essential for predicting the biological behavior and potential toxicity of nanomaterials. In existing literature reports, the primary exposure routes for nanomaterials in living organisms include inhalation, oral ingestion, dermal contact, intraperitoneal injection, and intravenous injection. Different exposure routes result in variations in their absorption and distribution patterns.

In studies on respiratory exposure, researchers primarily use methods such as inhalation, intratracheal instillation, and intranasal instillation to investigate the effects of NBNs on living organisms. To explore the differential metabolites and metabolic pathways in rat serum and further verify the potential mechanism of bile acid metabolism disorder after exposure to NiO NPs, Zhang et al. administered intratracheal instillation of NiO NPs (0.24 mg/kg body weight) to 16 male Wistar rats, twice a week for 9 weeks [37]. After exposure to NiO NPs, 21 differential metabolites related to bile acids (BAs), lipids, and phospholipid metabolic pathways were identified in the rat serum. The reduction in cholic acid and deoxycholic acid indicated that BAs metabolism was disrupted. The increased nickel content in the liver after exposure to NiO NPs resulted in the disruption of BAs, lipid, and phospholipid metabolic pathways in rats. Nishi et al. analyzed previous data on intratracheal injection of NiO NPs in rats, focusing on the retention of nickel in the lungs and the ratio of lung weight of each rat to the average lung weight of control rats [60]. The in vivo experimental results indicated that as the mass of NiO NPs in the lungs increased, the lung weight ratio tended to increase. The study results suggest that the intratracheally injected NiO NPs slowly dissolve in the phagolysosomes of alveolar macrophages (AM), with the resulting Ni^2+^ ions causing AM to transform into foam cells at one month and still inducing an inflammatory response at three months’ post-injection.

Nickel is known for causing allergies and having carcinogenic properties, with its cancer-causing potential depending on its chemical form, as only specific nickel compounds can penetrate cells. Jimenez-Lamana et al. conducted the first study on the cytotoxicity, cellular uptake, and molecular targets of Ni NPs in human skin cells, comparing them with other chemical forms of nickel [95]. The dose–response curves for Ni NPs obtained in cytotoxicity assays exhibited the linear behavior characteristic of genotoxic carcinogens. Exposure of keratinocytes to Ni NPs resulted in the release of Ni^2+^ ions and their accumulation in the cytosol. It was found that cells exposed to Ni NPs at doses corresponding to moderate mortality rates synthesized a 6 kDa nickel-binding molecule. To elucidate the health effects of 28-day repeated oral administration of NiO NPs, Dumala et al. conducted a toxicity study on albino Wistar rats, following the Organisation for Economic Co-operation and Development (OECD) test guidelines. A dose-dependent increase in the values of two transaminases was recorded in the liver and kidney tissue homogenates. Biodistribution studies indicated that the highest nickel content was found in the liver, followed by the kidneys [101]. Singh et al. administered Ni NPs (< 30 nm) to rats via gavage at a dose of 5 mg/kg of body weight over two exposure periods, 15 days and 30 days, respectively. The study results indicated that these NPs accumulated in the ovaries and disrupted steroidogenesis.

Abdulqadir et al. administered 20 nm Ni NPs to rats via intraperitoneal injection for 28 consecutive days [102]. Using transmission electron microscopy (TEM), they observed the localization of Ni NPs in the kidneys and their effects on cellular ultrastructure. The results showed that Ni NPs induced various ultrastructural changes in the rat kidneys. The NPs crossed the basement membrane through multiple pathways, entering the cytoplasm of proximal tubular (PT) cells, and also traversed the plasma membranes of adjacent PT cells. The internalization, accumulation, and induced ultrastructural changes of Ni NPs adversely affected kidney function in rats. There are also reports in the literature indicating that highly adsorptive Ni NPs cause severe damage to epithelial cells through physical contact with the cell surface and the generation of ROS, while absorbable ionic nickel affects cellular antioxidant responses by being absorbed into the body and transported to the liver [103].

For in vivo MRI, the NiSe@PDA NCs were injected into tumor-bearing mice via the tail vein [97]. The authors investigated the tissue distribution of tumors in mice at different times (8 h, 24 h, and 7 days) after the injection. The accumulation of NiSe@PDA NCs in the liver and spleen was decreased at 24 h post-injection. By 7 days post-injection, the accumulation of NiSe@PDA NCs in the liver and spleen was nearly negligible. The behavior of NBNs within the body, including how they are absorbed, distributed, and taken up by cells, can change based on the method of exposure. More detailed research on the absorption, distribution, and uptake of NBNs needs to be conducted in the future.

### Mechanisms of action

The mechanisms of toxicity for nanomaterials can be complex and multifaceted, involving various cellular and molecular processes. NBNs have toxic effects on cells, animals and humans and play toxic roles mainly through oxidative stress, apoptosis, DNA damage, genotoxicity, mitogen-activated protein kinase (MAPK) signaling pathway, HIF-1a pathway and other mechanisms. Some of the key mechanisms by NBNs may exert toxic effects are listed as follows.

#### Oxidative stress

Oxidative stress is a toxic mode of action in which cellular metabolism cannot keep up with the detoxification of ROS, such as peroxides, superoxides, and oxygen free radicals, which can damage biomolecules within cells [105]. Oxidative stress can be caused by an excess production of ROS or by a malfunction in the pathways that eliminate them. Nel et al. proposed that the primary mechanisms by which nanomaterials induce various biological toxic effects are the generation of ROS and the resultant oxidative stress response [106]. It has been reported that Ni NPs can induce apoptosis in HepG2 and A549 cells through oxidative stress injury, ultimately arresting the cells in the sub-G1 phase [107].Therefore, the primary mechanism of Ni NP toxicity is likely oxidative stress damage. This may be attributed to nickel’s ability to bind with amino acids, polypeptides, and proteins, thereby promoting the production of ROS [108]. Kong et al. discovered that Ni NPs decreased the activity of superoxide dismutase (SOD) and catalase in rats, while increasing the levels of ROS, malondialdehyde (MDA), and nitric oxide (NO) [109]. This indicates that upon exposure to Ni NPs, the levels of antioxidant enzymes SOD and calalase initially rise in an attempt to counteract ROS-induced damage. However, when the antioxidant response is insufficient to neutralize the ROS, the balance between ROS production and the antioxidant defense system is disrupted. This imbalance leads to a reduction in antioxidant enzyme levels, an increase in ROS levels, oxidative stress, and potentially cell death.Singh et al. investigate the role of oxidative stress in male reproductive toxicity induced by NiO NPs in rats. Results on lipid peroxidation (MDA, H_2_O_2_, and NO) and oxidative stress (GSH-px and catalase) thus studied in testes exhibited adverse effects of NiO NPs.

Moreover, Khan et al. synthesized magnetic NiFe_2_O_4_ NPs and assessed their potential adverse effects in rabbits. The results indicated an increase in oxidative stress levels and a reduction in antioxidant enzyme levels in the rabbits that were treated with the NPs [51]. In a separate study by Djebbi et al., it was observed that the antioxidant enzymatic activity significantly increased with the concentration and duration of exposure to NiO NPs, suggesting that NiO NPs could induce oxidative stress in C. ponticus even with short-term exposure [33]. Furthermore, Hussain et al. reported findings from a study involving male mice treated with NiO NPs, which showed elevated levels of blood urea nitrogen, increased SOD in the liver, elevated MDA in the liver, kidney, and heart, and decreased catalase activity in the heart and kidney [110]. Female mice treated with NiO NPs exhibited significantly reduced levels of serum albumin and total proteins, increased SOD in the lungs, and elevated MDA in the liver. Moreover, exposure to NiO NPs and microparticles (MPs) led to a notable increase in the production of ROS such as MDA, H_2_O_2_, and NO in the kidneys of rats. The reduced values of reduced GSH and enzymes, namely SOD, glutathione peroxidase (GSH-Px) and catalase, confirmed that ROS was the key factor in the nephrotoxicity induced by NiO NPs in rats.

#### Apoptosis and autophagy

**Fig. 4:**
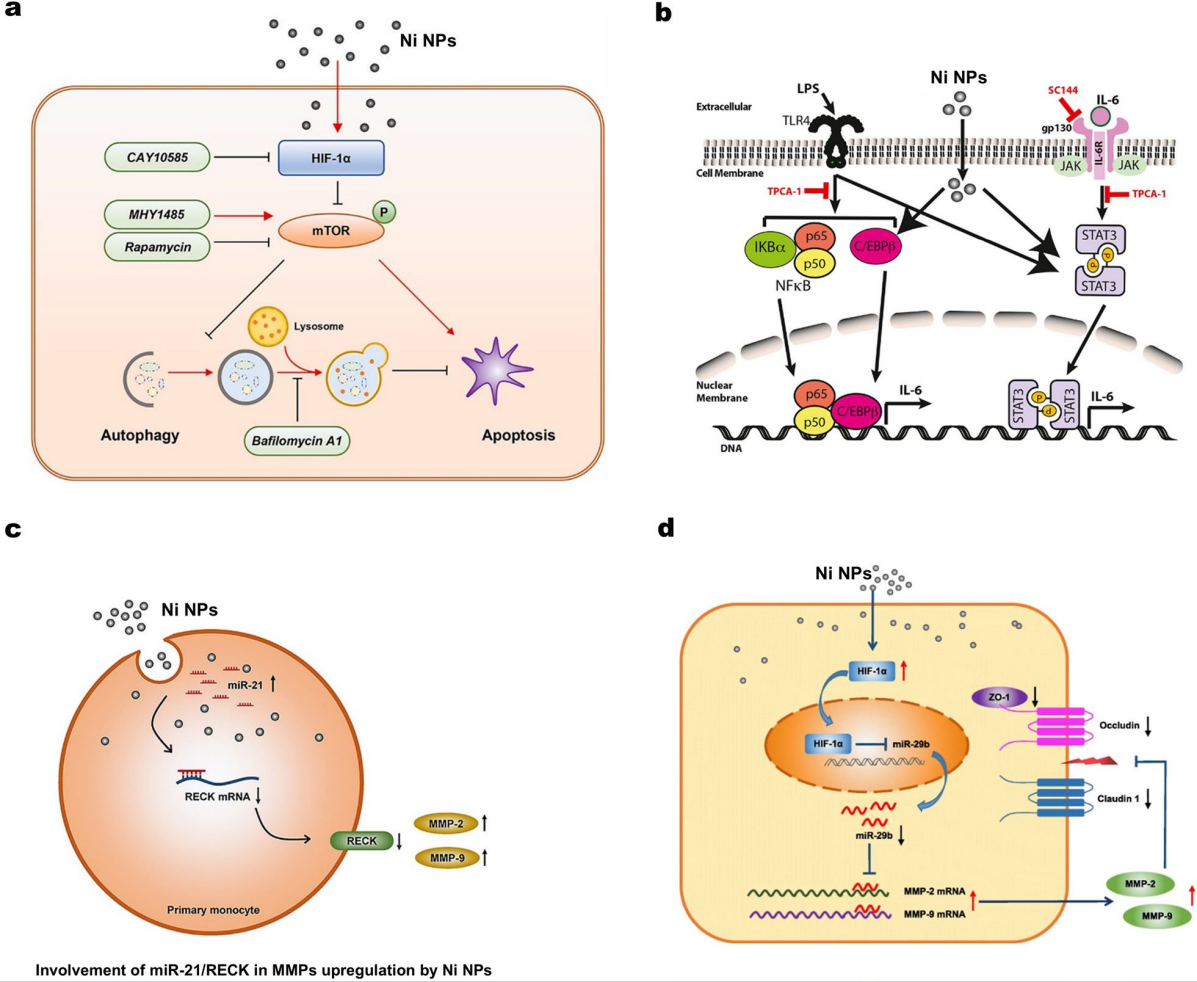
**a** Schematic graph and potential mechanisms of Ni NPs-induced autophagy and apoptosis in BEAS-2B cells. Reproduced with permission [87]. Copyright 2023, Elsevier. **b** Illustration of hypothetical mechanism of enhanced IL-6 production by LPS and Ni NPs in BEAS-2B cells in vitro. Reproduced with permission [104]. Copyright 2022, Elsevier. **c** The role of miR-21 in Ni NPs-induced MMP-2 and MMP-9 production in mouse primary monocytes. Reproduced with permission [40]. Copyright 2020, Elsevier. **d** Schematic and potential mechanisms of Ni NPs-induced dysregulation of tight junction-associated proteins in skin keratinocytes via HIF-1α/miR-29b/MMPs pathway. Reproduced with permission [30]. Copyright 2023, Elsevier

Apoptosis is a programmed and orderly cell death, characterized by cell shrinkage and fragmentation. Autophagy is a fundamental cellular process that involves the removal, degradation, or recycling of dysfunctional cellular components. Lysosomes, which are spherical organelles containing hydrolytic enzymes, play a crucial role in the autophagic process [111]. Wang et al. discovered that the neurobehavioral and neurodevelopmental disorders resulting from exposure to environmental NiO NPs are associated with ROS-mediated apoptosis and iron-mediated ferroptosis [36]. These two forms of programmed cell death are known to play significant roles in various central nervous system (CNS) injuries and diseases. The study also demonstrated that pharmacological interventions targeting the apoptosis and ferroptosis pathways using N-acetylcysteine (NAC) and deferoxamine (DFO), respectively, provided strong evidence linking apoptosis and ferroptosis to the neurotoxic effects induced by NiO NPs. Yuan et al. investigated the effects of Ni NPs on autophagy and apoptosis in human lung epithelial cell model (BEAS-2B), as well as the underlying mechanisms involved (Fig. 4a) [87]. Their results demonstrated that exposure to Ni NPs increased levels of both autophagy and apoptosis through the HIF-1α/mTOR signaling pathway. Interestingly, Ni NPs-induced autophagy appears to play a protective role against Ni NPs-induced apoptosis.

Moreover, Liu et al. found that intratracheal instillation of NiO NPs increased levels of pro-inflammatory cytokines, neutrophil and proteins in the bronchoalveolar lavage fluid (BALF) and triggered apoptosis and iron-deficiency in the lung tissue. Exposure to NiO NPs led to the upregulation of stress-induced transcription factor 3 (ATF3) in mouse lung tissue and BEAS-2B cells. Notably, BEAS-2B cells deficient in ATF3 exhibited reduced levels of apoptosis and ferroptosis upon exposure to NiO NPs [86]. In another study, yeast cells exposed to NiO NPs exhibit loss of cell viability, cytoplasmic outer leaflet phosphatidylserine exposure, nuclear chromatin agglutination, and DNA damage during protein synthesis, expression of apoptosis (RCD) [112]. Mitochondria are involved in the RCD process of apoptosis, which indicates that NiO NPs can induce apoptosis in yeast which is dependent on caspase and mitochondria.

#### Inflammation and fibrosis

Nanomaterials have been proven to enter the human body through various pathways, such as inhalation, skin contact, or ingestion [113]. Once inside the body, they may trigger inflammatory responses in cells and tissues, and prolonged exposure can even lead to fibrosis. Fibrosis is a pathological condition involving excessive growth of connective tissue, which results in damage to tissue structure and function. Many studies have shown that nickel-containing nanomaterials can cause inflammation and fibrosis in animals. You et al. employed an in vitro BEAS-2B cells to explore the intracellular signaling pathways involved in IL-6 production induced by LPS and Ni NPs (Fig. 4b) [104]. They also assessed the impact of sex hormones on IL-6 production triggered by Ni NPs and LPS in vitro. The study found that LPS and Ni NPs synergistically increased the expression of IL-6 mRNA and protein in BEAS-2B cells. TPCA-1, a dual inhibitor targeting IKK-2 and STAT3, effectively blocked this synergistic IL-6 increase, inhibited STAT3 activation, and reduced C/EBPβ levels. Conversely, SC144, an inhibitor of the IL-6 receptor component gp130, enhanced IL-6 production induced by LPS and Ni NPs.

In addition, Mo et al. investigated the impact of Ni NPs (nano-nickel), partially [O]-passivated nano-nickel (nano-nickel-p), and carbon-coated nano-nickel (nano-nickel-C) on the production of MMP-2 and MMP-9 by primary mouse monocytes, both in vitro and in vivo [40]. They also explored the underlying mechanisms involved. Their findings indicate that exposure of primary monocytes from wild-type mice to nano-nickel and nano-nickel-p results in the down-regulation of RECK, a direct target of miR-21. This suggests that the miR-21/RECK pathway plays a role in the production of MMP-2 and MMP-9 induced by nano-nickel (Fig. 4c). Jeong et al. [114] investigated the interaction between the lung microbiome and inflammatory responses in rats exposed to NiO NPs. Their study revealed that NiO NPs induced neutrophilic and lymphocytic inflammation in the rat lungs. Furthermore, they demonstrated that exposure to NiO NPs could alter the lung microbial composition. Specifically, they observed a higher presence of Burkholderiales in the NiO NP exposure groups compared to the control group one day after instillation. This dysbiosis in the lung microbiome was associated with acute lung inflammation.

Moreover, Zhan et al. [115] explored the role of long noncoding RNA (lncRNA) maternally expressed gene 3 (MEG3) in NiO NPs-induced collagen deposition. Male Wistar rats were intratracheally instilled with NiO NPs. They discovered that NiO NPs-induced rat pulmonary fibrosis was accompanied by the epithelial–mesenchymal transition (EMT) occurrence and MEG3 down-regulation in rat lung tissues. Their results indicated that MEG3 inhibited NiO NPs-induced collagen deposition by regulating transforming growth factor-β1 (TGF-β1)-mediated EMT process, which may provide some clues for insighting into the mechanisms of NiO NPs-induced pulmonary fibrosis.

#### Endoplasmic reticulum stress

Endoplasmic reticulum (ER) stress is a cellular condition that occurs when the demands of protein folding and processing in the ER exceed the cell’s capacity to handle them. Nanomaterials can induce ER stress, which occurs due to disruptions in intracellular calcium balance or protein misfolding [116]. ER stress can activate the unfolded protein response (UPR), impacting normal cellular functions. If the stress response is prolonged or excessive, it may lead to apoptosis and other pathological conditions, causing damage to tissues and organs. Therefore, understanding the mechanisms by which nanomaterials induce ER stress is crucial for assessing their biosafety and potential health risks. In a study by Zhou et al., the impact of Ni NPs on the liver of male C57/BL6 mice was investigated [49]. The mice were intraperitoneally injected with Ni NPs at doses of 10, 20, and 40 mg/kg/day for 7 and 28 days. The results showed that sustained exposure to Ni NPs led to a significant inflammatory response in the liver, upregulation of ER stress sensors such as inositol-requiring enzyme 1α (IRE1α), pancreatic ER kinase (PERK), and ATF6, and activation of apoptosis in liver cells. Chang et al. investigated the role of the ER stress pathway in NiO NPs-induced apoptosis in hepatocytes [117]. Male Wistar rats were intratracheally instilled with NiO NPs twice a week for 6 weeks. The upregulation of 78 kDa glucose-regulated protein and CCAAT/enhancer-binding protein homologous protein at both gene and protein levels suggested that NiO NPs triggered ER stress. Exposure to NiO NPs led to increased protein levels of IRE-1, p-IRE-1, X-box binding protein-1S, PERK, p-PERK, eukaryotic initiation factor-2α (eIF-2α), phosphorylated eIF-2α (p-eIF-2α), and caspases-12, −9, and −3. These findings indicate that NiO NPs can activate the ER stress-mediated apoptotic pathway.

#### Genotoxicity

Genotoxicity refers to the ability of certain substances or radiation to damage the genetic information within a cell, causing mutations, which can lead to cancer. Genotoxic agents include certain chemicals, environmental pollutants, radiation, and some naturally occurring compounds. As nanotechnology advances, Ni NPs are increasingly being utilized in various industries and biomedical applications. Several studies have investigated the genotoxic and carcinogenic effects of Ni NPs and have explored the underlying mechanisms involved. In one study, dose–response and time-response experiments were conducted using immortalized BEAS-2B cells to examine the impact of Ni NPs on DNA damage response (DDR)-associated proteins and the HIF-1α/miR-210/Rad52 pathway. Additionally, the study investigated DNA damage and disruption of the HIF-1α/miR-210/Rad52 pathway in vivo by intratracheally instilling 50 µg of Ni NPs per mouse. The research uncovered mechanisms by which Ni NPs induce malignant cell transformation, emphasizing the combined effects of Ni NPs-induced DNA damage and impaired DNA repair via the HIF-1α/miR-210/Rad52 pathway, likely contributing to genomic instability and subsequent cell transformation. These findings offer valuable insights into the molecular mechanisms underlying the genotoxic and carcinogenic properties of Ni NPs. In a separate study by Gamasaee et al., morphological and genotoxicity assessments demonstrated an increase in DNA fragmentation and the Bax/Bcl-2 mRNA expression ratio in lymphocyte cells following 24 h of treatment with NiO NPs. Akerlund et al. discovered that Ni NPs induced genotoxicity, evidenced by DNA damage resulting from single-strand breaks in six different types of mouse embryonic stem cells. The study found that Ni NPs exhibited a more pronounced genotoxic effect compared to NiO NPs and nickel chloride (NiCl_2_) [118].

#### MAPK signaling pathway

The mitogen-activated protein kinase (MAPK) signaling pathway is a crucial cellular mechanism that transmits signals from the cell surface to the nucleus in response to stimuli like growth factors, cytokines, and stress [119]. It involves a cascade of protein kinases leading to the activation of transcription factors that regulate gene expression. key MAPK pathways, such as ERK1/2, JNK, and p38, control essential cellular processes like proliferation, differentiation, apoptosis, and stress responses. Dysregulation of this pathway is linked to diseases including cancer, inflammatory disorders, and neurodegenerative diseases.

To elucidate the mechanisms by which NiO NPs induced pulmonary fibrosis, Tian et al. investigated the roles of TGF-β1, the MAPK pathway, and the balance between matrix metalloproteinases (MMPs) and tissue inhibitors of metalloproteinases (TIMPs) in NiO NPs-induced lung fibrosis [120]. They assessed the protein levels of TGF-β1, MAPKs, and MMPs/TIMPs using western blot analysis. The results indicated that NiO NPs exposure led to increased levels of hydroxyproline, collagen I, and TGF-β1, along with the activation of the MAPK pathway and an imbalance in MMPs/TIMPs, all in a dose-dependent manner. Additionally, they found that the MAPK signaling pathway was inhibited by a TGF-β1 inhibitor. Inhibitors of p38 MAPK and ERK1/2 mitigated the increases in Hyp and Col-I levels and corrected the MMPs/TIMPs imbalance. These findings suggested that the MAPK pathway and MMPs/TIMPs imbalance played a role in NiO NPs -induced excessive collagen formation.

Moreover, Saquib and colleagues were the first to elucidate the mechanism of in vivo toxicity of NiO NPs via oral administration [121]. To uncover the molecular mechanisms underlying cell death, they analyzed the translational activation of apoptotic proteins using western blotting. Following a 14-day exposure to NiO NPs at a dosage of 4.0 mg/kg body weight per day, there was a significant upregulation in the expression of p53 and MAPKAPK-2. The authors presented novel evidence that NiO NPs activate MAPKAPK-2 signaling to induce cell death. The p38 MAPK pathway plays a crucial role in NiO NPs-induced pulmonary inflammation and injury. In a study by Yang et al., Wistar rats were intratracheally instilled with NiO NPs suspensions at doses of 0.015, 0.06, and 0.24 mg/kg, administered twice a week for 9 weeks [122]. The researchers found that NiO NPs activated the p38 MAPK pathway and downregulated MEG3 both in vivo and in vitro. However, treatment with a p38 MAPK pathway inhibitor reversed the NiO NPs-induced changes in inflammatory cytokine expression levels. Furthermore, overexpression of MEG3 significantly inhibited the activation of the p38 MAPK pathway and the associated alterations in inflammatory cytokines caused by NiO NPs.

#### HIF-1α pathway

Hypoxia-inducible factor-1 (HIF-1) is a crucial transcription factor that plays a central role in the cellular response to low oxygen (hypoxia) conditions. HIF-1 is a heterodimer composed of two subunits HIF-1α and HIF-1β. HIF-1α is oxygen-sensitive subunit that is stabilized under hypoxic conditions, allowing it to dimerize with HIF-1β and activate hypoxia-responsive genes. The HIF-1α pathway activated by nanomaterials raises several safety concerns [123, 124]. These include promoting cancer, inducing chronic inflammation, contributing to fibrosis, causing metabolic dysregulation, and impacting cardiovascular health.

As an illustration, Pietruska et al. observed that Ni NPs can activate the HIF-1α pathway, which subsequently leads to the malignant transformation of cells and tumor formation [125]. In comparison to nickel microparticles (Ni MPs) or soluble nickel, Ni NPs are more effective at activating the HIF-1α hypoxia signaling pathway. Under hypoxic conditions, HIF-1α translocates from the cytoplasm to the nucleus, where it binds with HIF-1β to form the active HIF-1 protein. This activation triggers hypoxia-responsive genes, resulting in the malignant transformation of cells. Recent studies demonstrated that exposure to Ni NPs leads to nuclear accumulation of HIF-1α in BEAS-2B cells, whereas TiO_2_ NPs does not have this effect [87, 123]. Qian et al. demonstrated that HIF-1α and TGF-β1 work together synergistically to promote pulmonary fibrosis induced by NiO NPs. The interaction between these two factors is a crucial mechanism underlying the development of pulmonary fibrosis [126]. Zhang et al. conclude that hypoxia stress played a pivotal role in NiO NPs induced hepatoxicity. NiO NPs triggered hypoxia by up-regulation of HIF-1α and miR-210 in HepG2 [30]. Zhang et al. demonstrated that exposure of human epidermal keratinocytes to Ni NPs results in increased transcription and activity of MMP-2 and MMP-9, along with an imbalance in tight junction-related proteins. Their findings revealed that the down-regulation of miR-29b expression, triggered by Ni NPs, occurs due to the accumulation of HIF-1α in the nuclei, also induced by Ni NPs. The disruption of tight junction proteins in skin keratinocytes caused by Ni NPs is mediated through the HIF-1α/miR-29b/MMPs pathway (Fig. 4d). These insights contribute to a deeper understanding of the skin toxicity associated with Ni NPs exposure.

### Biotransformation, clearance, and excretion

The biotransformation of NBNs in animals involves their chemical and physical modifications post-exposure, which can significantly affect their bioavailability, distribution, toxicity, and ultimate fate within the organism. The biotransformation processes can influence the excretion and clearance of NBNs. Solubilized ions and smaller degradation products may be more readily excreted through renal filtration or biliary excretion, while larger aggregates or intact NPs may be sequestered in organs or excreted more slowly. Understanding the biotransformation, clearance, and excretion of NBNs is important for toxicity assessment and risk management.

The localization of Ni NPs in the kidneys of rats and their effects on cellular ultrastructure suggested that these particles could enter renal tubular cells through various pathways, leading to significant ultrastructural changes [102]. This indicated that Ni NPs could be absorbed by the kidneys, potentially affecting renal function. Furthermore, there was a significant correlation between the concentration of nickel in urine and the total urine volume, particularly after long-term exposure [127]. This confirmed the presence of Ni NPs in urine and highlighted their potential impact on human health.

The excretion of nickel in urine appeared to be closely related to urine volume. Low levels of oral exposure (300 ppm Ni) increased nickel excretion in urine, while higher doses (1200 ppm) resulted in a substantial rise in nickel concentration in urine. This suggested a strong relationship between nickel excretion and urine volume [128]. Additionally, the presence of nickel in urine was associated with changes in other biochemical parameters, such as creatinine, uric acid, and urea. These findings indicated that variations in nickel concentration in urine could reflect exposure to specific chemical substances.

A case in point is the analysis of the metabolic products of NiO NPs within the body. Zhang et al. found that after exposure to NiO NPs, 21 differential metabolites related to alkaline amino acid, fat, and phospholipid metabolic pathways were identified in the serum of rats. NiO NPs may cause a decrease in serum bile acids (BAS) by downregulating the synthases involved in BAS metabolism and overexpressing transmembrane proteins and detoxification enzymes, thereby affecting hepatobiliary clearance [37]. Hu et al. collected urine and feces from mice over a seven-day period following the intravenous injection of NiSe@PDA nanocomposites [97]. Their findings suggested that these nanoagents are primarily excreted from the body via feces. The significant accumulation of the nanoagents in the liver and spleen post-injection indicates that the reticuloendothelial system plays a key role in their clearance, with excretion occurring mainly through feces.

Moreover, Tammam et al. found that the serum urea and creatinine levels in the NiO NPs exposure group had significantly elevated, indicating a high incidence of renal dysfunction. This suggested that NiO NPs underwent biotransformation in the liver, were encapsulated by the reticuloendothelial system, and were excreted by the kidneys. The glomeruli in the kidneys had 3.5 nm slit pores, which allowed NiO NPs of this size to accumulate within the renal tissue, leading to hyperactivity of the renal tubules and altered glomerular filtration throughout the clearance process. This indicated that the kidneys were a crucial pathway for the clearance and excretion of these NPs in the body [129]. The excretion of NBNs in the kidneys and urine was complex, involving the biological effects of these nanomaterials, changes in cellular ultrastructure, and fluctuations in nickel concentration in urine. These results underscored the importance of long-term monitoring and assessment of NBNs to better understand their potential impact on human health.

### Toxic effects by organ system

**Fig. 5:**
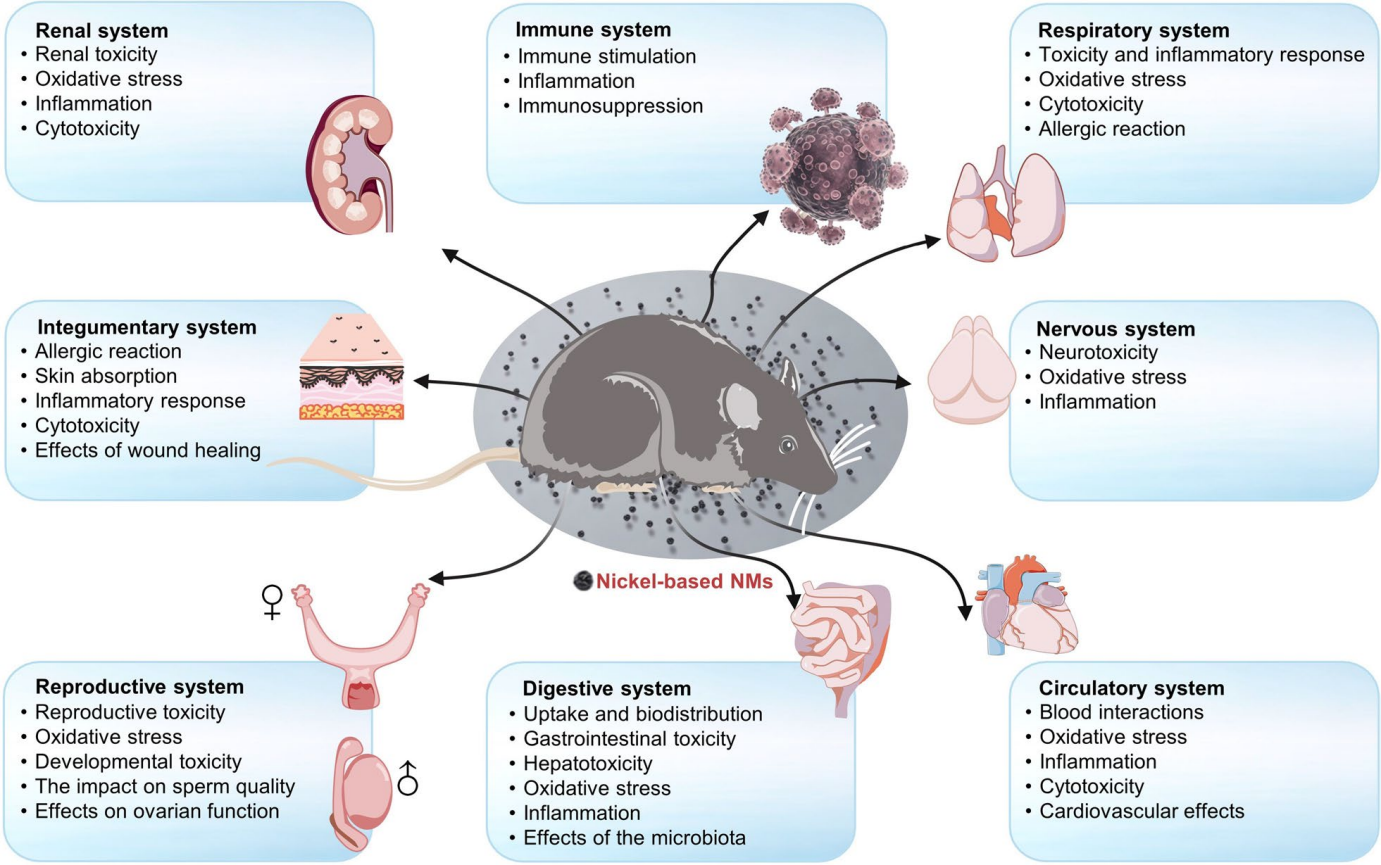
The main toxic effects of NBNs on various organ systems

Nanomaterials can cause toxic effects in multiple organ systems. Inhalation can damage the respiratory system, while the cardiovascular system may experience oxidative stress and inflammation, leading to hypertension. The liver and kidneys can suffer from toxicity due to accumulation. Some nanomaterials cross the blood–brain barrier, causing neurotoxicity, and ingestion can alter gut microbiota, leading to intestinal inflammation. We have summarized the main toxic effects of NBNs on various organ systems (Fig. 5). Understanding these effects is vital for assessing nanomaterial safety in various applications. We also have summarized various animal models to evaluate the biotoxicity of NBNs (Table 2). Below are the toxic effects of NBNs on several major organ systems.

**Table 2:** In vivo effects of NBNs on different animal types

NPs Type	Animal	Sex	Route	Findings	Refs.
NiO	Rats	Male	Oral gavage	Oxidative damage and physiological disruption in rats’ liver and kidney	[129]
NiO	Rats	Male	Oral gavage	Reproductive toxicity	[57]
NiO	Rats	Male and female	Oral gavage	Adverse alteration of the biochemical profile and histological damage	[101]
NiO	Rats	Female	Intratracheal	Neutrophilic and lymphocytic lung inflammation and alteration of microbial composition	[114]
Ni	Rats	Male	Intraperitoneal	Induce various cellular ultrastructural changes in the kidneys	[102]
NiO	Rats	Male	Intraperitoneal	Alter biological indices, impair antioxidant status, and induce tissue damage	[149]
Ni	Rats	Male	Intraperitoneal	Reproductive toxicity	[74]
Ni	Mice	Male	Intragastric	Reproductive toxicity	[42]
Ni	Mice	Male	Intratracheal	Reproductive toxicity	[146]
Ni	Mice	Female	Oral gavage	Intestinal epithelial tissue damage and induction of allergic reactions	[47]
Ni	Mice	Male and female	Oropharyngeal aspiration	Induce mild monocytic lung and neutrophilic inflammation	[45]
Ni, Ni-C, Ni–P	Mice	Male	Intratracheal	Induce acute lung inflammation and injury	[39]
Ni	Mice	Male	Intraperitoneal	Induce liver injury and disturbance of lipid metabolism	[49]
NiFe_2_O_4_	Rabbits	NR	Via ear vein	Induce adverse effects on hematology, serum biochemistry, oxidative responses, and microscopic alterations in multiple visceral tissues	[51]
NiO	Mussel	NR	Soaking	Oxidative stress and cytotoxicity	[150]
NiO	Zebrafish	Male and female	Soaking	Neurotoxicity and developmental toxicity	[36]
Ni	Zebrafish	NR	Soaking	Cause tissue damage in the gills, digestive tract, and liver	[103]
Ni	Fishes (Labeo rohita)	NR	Soaking	Behavioral and metabolic toxicity pathological lesions in liver and kidney	[135]
NiO	Fishes (Heteropneustes fossilis)	NR	Soaking	Induce substantial toxicity, changes in the haematological parameters and haematological indices	[72]
NiO	Beetle	Female	Ventrocaudal area injection	Cause DNA damage and a high degree of cellular toxicity in the ovarian cells	[151]
NiO	Beetle	NR	Ventrocaudal area injection	Histological and ultrastructural changes in the midgut tissues	[152]
Ni	Soil ecotox model	Hermaphrodite	Soaking	Increase proteolysis, apoptosis, inflammatory response, and neurotoxicity	[153]
NiO	Eisenia fetida	Hermaphrodite	Soaking	Biochemical, histopathological, reproductive, developmental and genic toxicity	[154]
NiO	Copepods	Female	Soaking	Reproductive, metabolic and neural toxicity	[33]

#### Respiratory system

Nanomaterials can have significant effects on the respiratory system. Inhalation of these particles can lead to inflammation, oxidative stress, and damage to lung tissue [130]. This can result in conditions such as bronchitis, asthma, and fibrosis. Nanomaterials can also impair lung function and exacerbate existing respiratory diseases. Long-term exposure may increase the risk of developing chronic respiratory conditions and even lung cancer.

In human lung cells, exposure to NBNs resulted in a decrease in cell viability and triggered apoptosis in a dose-dependent manner. The presence of oxidative stress was evidenced by elevated levels of markers such as dichlorofluorescein (DCF), GSH, MDA, and lactate dehydrogenase (LDH) [107, 131]. The inflammatory response was indicated by an increase in interleukin-8 (IL-8) release and COX-2 expression. Moreover, extracellular concentrations of glucose, lactate, phenylalanine, histidine, and tyrosine showed a time- and dose-dependent increase. Additionally, DNA damage and ER stress were observed in the lung cells exposed to NBNs. Apoptosis was linked to the activation of ATF3 [86], p53, Bax, caspase-3, Bcl-2 pathways [38], and the HIF-1α/mTOR signaling axis. Interestingly, some cells evaded apoptosis by activating autophagy [87].

The respiratory system is primarily affected when organisms inhale NBNs, which can deposit in the respiratory tract and lungs, potentially causing physical damage. Larger particles may be blocked in the upper respiratory tract, while smaller particles can reach the alveoli, leading to localized inflammation, fibrosis, and allergic reactions [110]. For example, intratracheal injection of NiO NPs in mice can increase levels of pro-inflammatory cytokines, neutrophils, and bronchoalveolar lavage proteins, as well as induce apoptosis and iron accumulation in lung tissue [86].Research has also shown that NiO NPs cause neutrophil and lymphocyte inflammatory responses in rat lungs. In this study, acute pulmonary inflammation in rats also led to dysbiosis of the lung microbiome [114].NiO NPs induce lung fibrosis in rats by activating TGF-β1, a process that is accompanied by EMT and downregulation of MEG3 in lung tissue [115].

In another study, rats were intratracheally administered 0.2 mg of NiO NPs suspended in distilled water and sacrificed at intervals ranging from 3 days to 6 months. The concentrations of 21 cytokines associated with inflammation, fibrosis, and allergy were evaluated in the lungs of the exposed rats. The results revealed persistent infiltration of alveolar macrophages in the group exposed to NiO NPs. The expression of macrophage inflammatory protein-1α (MIP-1α) in lung tissue and BALF continued to increase over time. Additionally, IL-1α and IL-1β in lung tissue, as well as monocyte chemoattractant protein-1 (MCP-1) in BALF, exhibited transient increases following exposure to NiO NPs. These findings indicate that the aggregates of NiO NPs have a sustained inflammatory effect [132]. Ni NPs also cause severe and persistent lung inflammation and fibrosis, with miR-21 playing a significant role in nanoparticle-induced pulmonary toxicity [48].

Additionally, there are gender differences in the induction of lung diseases by NBNs. For acute exposure, male and female mice were administered a single dose of Ni NPs (with or without lipopolysaccharide (LPS)) via oropharyngeal aspiration and were sacrificed 24 h later. For subchronic exposure, mice received six doses of Ni NPs (with or without LPS) over a period of 3 weeks before being sacrificed. Subchronic exposure to Ni NPs in female mice induced STAT1 in lung tissue, whereas this was not observed in males. In males, acute exposure to Ni NPs and LPS resulted in a more pronounced induction of IL-6 mRNA in the liver. On the other hand, subchronic exposure to Ni NPs led to a higher induction of monocyte chemoattractant protein-1 (CCL2) mRNA in the liver. These findings suggest that males exhibit a higher susceptibility to acute pulmonary inflammation, characterized by neutrophilia and increased levels of CXCL1 and IL-6/STAT3 signaling. In contrast, their susceptibility to subchronic pulmonary inflammation involves increased monocyte infiltration with elevated levels of CXCL1 and CCL2. The differences in susceptibility between males and females in these inflammatory responses may be influenced by the involvement of STAT transcription factors.

#### Circulatory system

Nanomaterials can significantly impact the circulatory system [133]. They may induce oxidative stress and inflammation, leading to endothelial dysfunction and vascular damage. Exposure to nanomaterials can also result in altered blood pressure, thrombosis, and impaired blood flow. Additionally, these materials can interact with blood components, potentially causing hemolysis and affecting coagulation pathways. Understanding these effects is crucial for evaluating the safety of nanomaterials in medical and industrial applications. Nickel-titanium (NiTi or Nitinol) alloys are commonly utilized in various biomedical applications, including the manufacturing of cardiac, peripheral vascular, and fallopian tube stents. However, there are notable biocompatibility concerns associated with Ni^2+^ ions and nano/micron-sized alloy particles from these metal implants. Studies have demonstrated that Ni^2+^ ions and NiTi NPs can trigger the expression of pro-inflammatory and pro-angiogenic cytokines/chemokines in vitro in human endothelial and monocyte cell lines [44]. These findings raise concerns about potential mechanisms of stent failure, as inflammation and angiogenesis can contribute to implant failure, especially in the context of stents. To investigate the role of nickel and NiTi NPs in promoting angiogenesis in vivo, a 1cm silicone vascular reactor was subcutaneously implanted in athymic (T-cell deficient) nude mice. It was found that NiTi NPs exhibited significant angiogenic properties, while Ni^2+^ ions were less pronounced.

Researchers have developed a simple, environmentally friendly, and economical green chemistry method to prepare NiO NPs using the fermented liquid of fresh Rhamnus triquetra (RT) leaves. The RT leaf culture liquid is used as a strong reducing, capping, and stabilizing agent in the formation of RT-NiO NPs. They conducted various in vitro bioactivity tests on RT-NiO NPs using red blood cells, demonstrating unique biosafety and biocompatibility potential [134]. In a study, researchers evaluated the toxicological effects of exposing fish to Ni NPs (43 nm) for 21 days at a concentration of 25 mg/L. They observed that in fish, the total number of red blood cells was lower in all fish treated with NPs. A significant decrease in growth and hemoglobin was observed in fish treated with Ni NPs [135].

For example, Martínez-Rodríguez et al. have studied the in vitro toxicity of NiFe_2_O_4_ NPs on human red blood cells and peripheral blood mononuclear cells. To assess their potential toxicity, in vitro studies were conducted with different concentrations of nickel-zinc ferrite (Ni_0.5_Zn_0.5_Fe_2_O_4_) NPs. The toxicity within the human body was evaluated by determining hemolysis of red blood cells, measuring total protein content, and analyzing the activities of catalase and glutathione-S-transferase. The results indicated that Ni_0.5_Zn_0.5_Fe_2_O_4_ NPs cause hemolysis. No significant changes in the viability of human peripheral blood mononuclear cells are observed after treatment with NiFe_2_O_4_ NPs in vitro [136].

Specifically, Gamasaee et al. evaluated the effects of NiO NPs on the structural changes, heme degradation, and aggregation of hemoglobin (Hb). NiO NPs can induce morphological changes in human lymphocytes and the expression of Bax/Bcl-2 mRNA, as well as cause the displacement of aromatic residues and heme groups, and the production of pre-aggregates. Furthermore, NiO NPs lead to the degradation of heme and the amorphous aggregation of hemoglobin, as well as cause tertiary conformational changes in hemoglobin and heme displacement. Molecular simulation studies also confirmed the structural changes in hemoglobin and heme deformation caused by NiO NPs. Additionally, morphological and genotoxicity analyses showed that after 24 h of treatment with NiO NPs, there was an increase in DNA fragmentation and the expression rate of Bax/Bcl-2 mRNA in lymphocytes. In summary, this study indicates that NiO NPs may affect biological media.

#### Digestive system

Nanomaterials can impact the digestive system in several ways [137]. Ingestion of these particles can lead to gastrointestinal inflammation, oxidative stress, and disruption of the gut microbiota. This may result in symptoms such as nausea, diarrhea, and abdominal pain. To investigate the toxicity of orally administered NiO NPs, human intestinal epithelial cells (Caco-2) were utilized to preliminarily assess the toxic effects at the cellular level. The findings revealed that NiO NPs induced a 50% reduction in cell viability at a concentration of 351.6 µg/mL and caused DNA damage and oxidative stress at concentrations ranging from 30 to 150 µg/mL. Apoptosis appears to be the primary mechanism of cell death in intestinal cells exposed to NiO NPs [138].

There have been some studies on the effects of NiO, Ni NPs, and NiFe_2_O_4_ NPs on the digestive system, using animal models including mice, fish models, and rabbits [49, 51, 139]. The exposure routes for these NBNs include oral administration, intraperitoneal injection, and tracheal instillation. After oral administration of these materials, the liver and kidneys are significantly affected. One study showed that in rats, oral intake of NiO NPs resulted in a dose-dependent increase in the levels of two transaminases in liver and kidney tissue homogenates. SOD activity significantly decreased, while catalase activity increased. Additionally, there was a dose-dependent decrease in GSH levels in rats, indicating the production of ROS and oxidative stress. NiO NPs adversely altered the biochemical characteristics of the rats [101].In another study, BALB/c mice that were orally administered Ni NPs exhibited intestinal epithelial tissue damage, elevated serum levels of IL-17 and IL-1β, and increased nickel accumulation in the liver and kidneys [47]. In addition to mice models, a study evaluated the hazards of NiO NPs on the gills and liver of Indian catfish [139]. Nickel accumulation, lipid peroxidation, antioxidant enzyme activity, liver enzyme activity, and immunohistochemistry were analyzed in gill and liver tissues. The results showed an increase in nickel accumulation in the tissues of exposed fish. Lipid peroxidation and activity of different antioxidants (except for SOD) increased after exposure. Changes in liver enzyme activity and Na^+^/K^+^ ATPase activity were also observed. Their research indicates that NiO NPs have a detrimental effect on the gills and liver tissues of fish, and therefore, wastewater containing these NPs should be treated before being released into water bodies.

#### Immune system

Nanomaterials can significantly affect the immune system [140]. They can trigger immune responses, leading to inflammation and the activation of various immune cells such as macrophages, neutrophils, and lymphocytes. This activation can result in the production of cytokines and other inflammatory mediators. The long-term impact of nanomaterial exposure on the immune system can potentially lead to chronic inflammatory conditions, autoimmune diseases, or altered immune responses. Morimoto et al. examined the cytokines associated with pulmonary diseases induced by well-dispersed NiO NPs (~ 26 nm) [132]. Rats were intratracheally administered 0.2 mg of NiO NPs suspended in distilled water and were euthanized within 3 days to 6 months. The concentrations of 21 cytokines were measured in the lungs, including those related to inflammation, fibrosis, and allergies. Persistent infiltration of alveolar macrophages was observed in the NiO exposure group. The expression of macrophage inflammatory protein-1α in lung tissue and BALF continuously increased, while IL-1α, IL-1β in lung tissue, and monocyte chemoattractant protein-1 in BALF showed transient increases. These studies indicate that the nanoclusters of NiO NPs have a persistent inflammatory effect, and the transient increase in cytokine expression and the continuous increase in cysteine-cysteine chemokines are associated with persistent lung inflammation.

It is worth mentioning that Jeong et al. studied the effects of NiO NPs on lung inflammation and investigated whether the NLRP3 inflammasome is involved in NiO NPs-induced lung inflammation and damage [141]. The study confirmed that NiO NPs exposure led to sustained lung inflammation, accompanied by inflammatory cell infiltration, alveolar protein deposition, and cytokine secretion. Furthermore, NiO NPs were found to significantly increase the expression of NLRP3, along with the overexpression of the active form of caspase-1 (P20) and the secretion of IL-1β in vivo. The secretion of IL-1β induced by NiO NPs was partially inhibited by co-treatment with a caspase-1 inhibitor in macrophages. Knockdown of NLRP3 using siRNA completely attenuated NiO NPs-induced cytokine release and caspase-1 activity in macrophages in vitro. Additionally, the activation of the NLRP3 inflammasome by NiO NPs was shown to be dependent on particle uptake and ROS production. Overall, the findings of this study suggest that the NLRP3 inflammasome plays a role in the induction of lung inflammation by NiO NPs.

#### Nervous system

Nanomaterials can significantly impact the nervous system. Some can cross the blood–brain barrier, causing neuroinflammation, neurotoxicity, oxidative stress, and neuronal damage, which impair neural function. Exposure may disrupt neurotransmitter levels and signaling pathways, potentially contributing to neurodegenerative diseases like Alzheimer's and Parkinson's. In microglial cells, NiO NPs significantly increased the cytotoxicity of aged α-synuclein amyloid protein [142]. The presence of NiO NPs amplified the cytotoxic effects of aged α-synuclein amyloid protein by promoting the production of high levels of ROS, inactivating SOD and catalase, and inducing inflammation, as evidenced by elevated levels of tumor necrosis factor-alpha (TNF-α), IL-1, and IL-1β. Additionally, apoptosis was markedly increased in the EOC 13.31 mouse microglial cell line. The accelerating effects of NiO NPs on the fibrillation of α-synuclein amyloid protein and the associated neurotoxicity are indicative of markers for Parkinson’s disease.

**Fig. 6:**
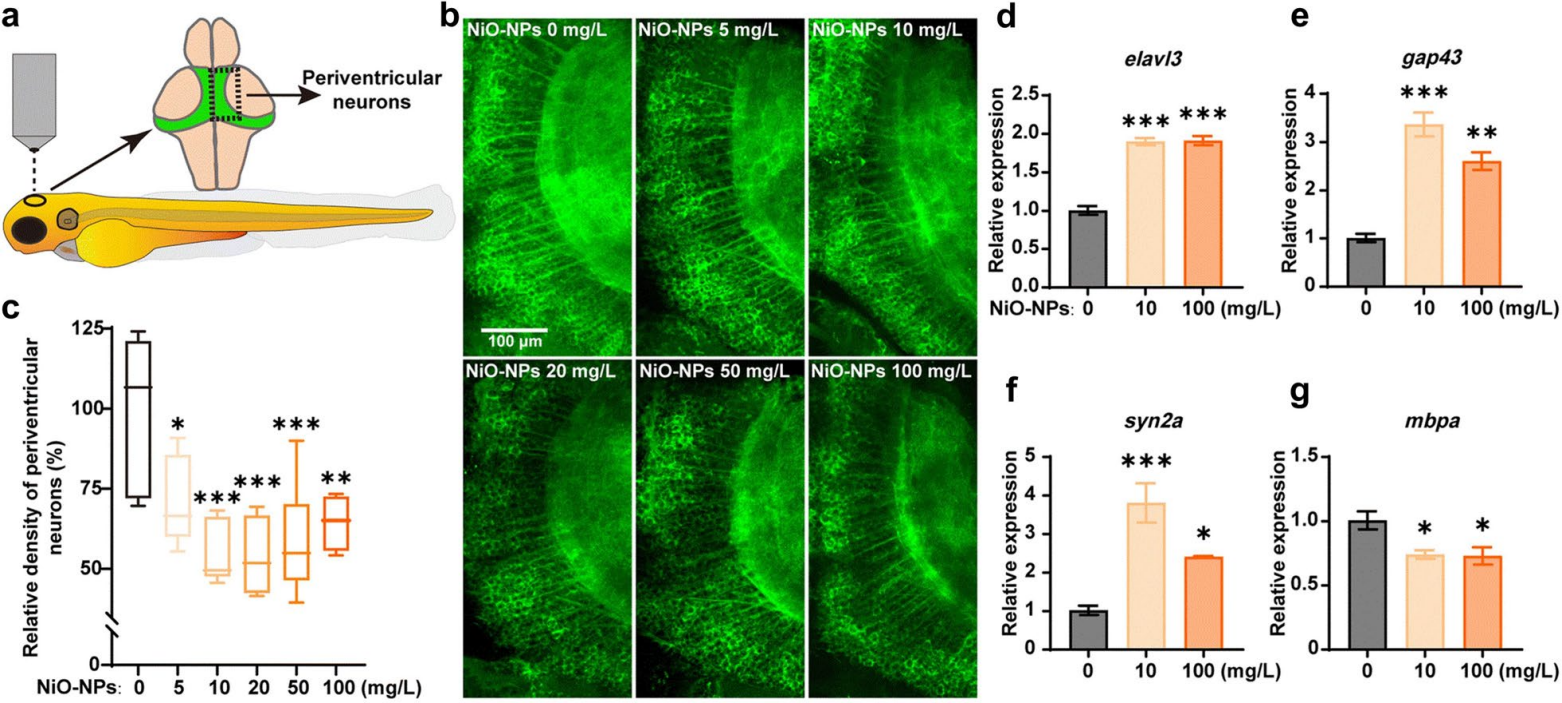
NiO NPs exposure affects neurogenesis of zebrafish larvae. **a** Scheme of the tectal periventricular layer (PVL) region observed in this study.** b** Representative fluorescent images of the tectal PVL region in NiO-NPs exposed *Tg* (*HuC: EGFP*) zebrafish larvae. **c** The relative density of periventricular neurons (PVN) in NiO-NPs exposed zebrafish larvae. **d–g** The relative expression of neurogenesis/neurodevelopment related genes. Reproduced with permission [36]. Copyright 2023, Elsevier

Previous research has indeed indicated that NPs have the potential to enter the CNS and elicit a range of adverse effects. Several animal models have been used to assess the impact of NBNs on the nervous system, including rats, zebrafish, and Enchytraeus crypticus (Oligochaeta). Wang and colleagues investigated the neurotoxic effects induced by NiO NPs and explored the potential mechanisms [36]. The researchers utilized the zebrafish model system to conduct genetic analysis, in vivo neural imaging, and neurobehavioral assessments to investigate the impact of early exposure to environmentally relevant concentrations of NiO NPs on the embryonic development, neurobehavior, and neural development of zebrafish larvae (Fig. 6). Through their study, they aimed to explore the potential effects of NiO NPs exposure on various aspects of zebrafish development and behavior. This study elucidated the potential cellular and molecular mechanisms of NiO NPs induced neurotoxicity, which is of great significance for researchers to understand the physiological damage effects of other environmental nanopollutants and for drug treatment.

#### Renal system

Nanomaterials typically have a significant impact on kidney function. They can accumulate in the kidneys, leading to oxidative stress, inflammation, and cellular damage. This may result in impaired kidney function, including reduced glomerular filtration rate and tubular dysfunction. Prolonged exposure to nanomaterials can potentially cause chronic kidney disease and exacerbate existing renal conditions. Singh et al. conducted a comparative and time-dependent study on the effects of NiO NPs and NiO MPs on the kidneys of male Wistar rats over intervals of 15 and 30 days [59]. They investigated changes in kidney morphology and function, as well as the impact on antioxidant enzyme activity. Both NiO NPs and MPs significantly increased the production of ROS in rat kidneys, including MDA, H_2_O_2_, and NO. The decreased levels of GSH and antioxidant enzymes (SOD, GSH-Px, and catalase) confirmed the role of oxidative stress in NiO NPs-induced nephrotoxicity. The study results suggest that the nephrotoxicity of NiO NPs is greater than that of nickel metal ions, with ROS being the key factor causing kidney toxicity in rats.

As an illustration, Abdulqadir et al. carefully examined the anticipated adverse effects of Ni NPs of different sizes on the rental units of rat kidneys [61]. They exposed the experimental animals to Ni NPs of three sizes (20 nm, 40 nm, and 70 nm) intraperitoneally every day. The most common histopathological features in the Ni NPs treatment groups were inflammatory cell infiltration with leukocytes and degeneration of proximal renal tubule cells, indicating Ni NPs-induced nephrotoxicity. The levels of MDA were significantly elevated in all Ni NPs treatment groups, with significant increases in tissue SOD and serum creatinine. Furthermore, a significant increase in p53 positive renal tubule cells was detected in the Ni NPs treatment groups compared to the control group.

#### Integumentary system

Nanomaterials can significantly impact the integumentary system, which includes the skin, hair, and nails. When applied topically or through occupational exposure, nanomaterials can penetrate the skin barrier, potentially causing oxidative stress, inflammation, and cellular damage. This can lead to conditions such as dermatitis, allergic reactions, and impaired wound healing. Despite the well-known allergenicity and carcinogenicity of nickel, its molecular mechanisms remain uncertain, necessitating research at the molecular level [95]. The carcinogenicity of nickel is known to depend on its chemical form, as only certain nickel compounds can enter cells. The damage to the skin caused by NBNs is also a matter of concern. Jimenez-Lamana et al. conducted a pioneering study to investigate the cytotoxicity, cellular uptake, and molecular targets of Ni NPs in human skin cells, comparing them with other chemical forms of nickel. In their research, the dose–response curve of Ni NPs in cytotoxicity tests exhibited a linear behavior characteristic of genotoxic carcinogens. Exposure of keratinocytes to Ni NPs led to the release of Ni^2+^ ions and their accumulation in the cytosol. Furthermore, the study revealed that cells exposed to Ni NPs at a dose equivalent to the median lethal dose synthesized a 6 kDa nickel-binding molecule, which was identified as the tumor-associated p63 regulated gene 1 protein. This finding sheds light on the molecular mechanisms underlying the cellular response to Ni NPs exposure and highlights the potential implications for skin cell health and function. An early study indicated that applying Ni NPs to the skin surface leads to an increase in nickel content within the skin and a significant permeation flux through the skin, which is even higher when a damaged skin protocol is employed [95].

The effects of NBNs on the integumentary system also extend to the treatment of skin-related cancers. Since the toxic effects of NiO NPs on human melanoma cell lines at the cellular and molecular levels have not yet been clearly elucidated, Rahimi et al. examined the in vitro cytotoxicity of NiO NPs on the mitochondria of malignant skin melanoma (MSM) [143]. The results showed that, compared to non-cancerous mouse skin mitochondria, NiO NPs significantly increased the levels of ROS, lipid peroxidation, and mitochondrial membrane potential, and decreased the activity of succinate dehydrogenase, GSH levels, and ATP content in the mitochondria of the melanoma mouse model skin. Additionally, some nanomaterials exhibit minimal cytotoxicity to skin cells and have potential applications in the treatment of skin-related diseases. For example, literature reports indicate that NiFe_2_O_4_ NPs are non-toxic to normal human dermal fibroblasts (HDF) and mouse melanoma (B16-F10) cells within a concentration range of 0 to 1 mg/mL [144]. The study by Rabbani et al. indicates that NiFe_2_O_4_ (NF) and Zn–NiFe_2_O_4_ (ZNF) NPs are promising candidates for antimicrobial and wound healing nanomedicines [145]. These NF and ZNF NPs were not cytotoxic to HDF cells at concentrations of 250 µg/mL and 125 µg/mL or below, respectively. Furthermore, it was observed that at these safe concentrations, fibroblast cells significantly proliferated over time.

#### Reproductive system

Ni NPs have been found to affect reproductive and embryonic toxicity. Ni NPs can significantly reduce the body weight, serum testosterone levels, and daily sperm production in Sprague Dawley rats, while the testicular index, nickel accumulation, and histological changes in testicular tissue increase with the dose of Ni NPs [74]. Liu et al. illustrated that dynamin-related protein 1 (Drp1)-mediated mitochondrial division and PTEN-induced putative kinase 1 (Pink1)/Parkin-mediated mitochondrial autophagy are key players in the male reproductive toxicity induced by Ni NPs [42]. In this process, Drp1 and Pink1/Parkin engage in an interaction cycle that accelerates the onset of cell apoptosis. This study highlights the intricate interplay between mitochondrial dynamics, mitophagy, and cell death pathways in the male reproductive toxicity associated with Ni NPs exposure. There are time-dependent differences in the toxicity of Ni NPs and MPs on rat ovaries. Nanoscale nickel accumulates in the ovaries, affecting the synthesis of steroids [56].Kong et al. reported that the rate of sperm deformities and serum levels of reproductive hormones significantly increased with rising concentrations of Ni NPs [146]. Testicular spermatogenic cells exhibited damage, and there was a notable increase in the number of apoptotic cells. Additionally, the expressions of crucial proteins (Drp1, Pink1, and Parkin) associated with mitochondrial fission/autophagy in testicular tissues were elevated following exposure to Ni NPs. These findings suggest that mitochondrial damage may play a pivotal role in the reproductive toxicity observed in male mice following intratracheal instillation of Ni NPs.

**Fig. 7:**
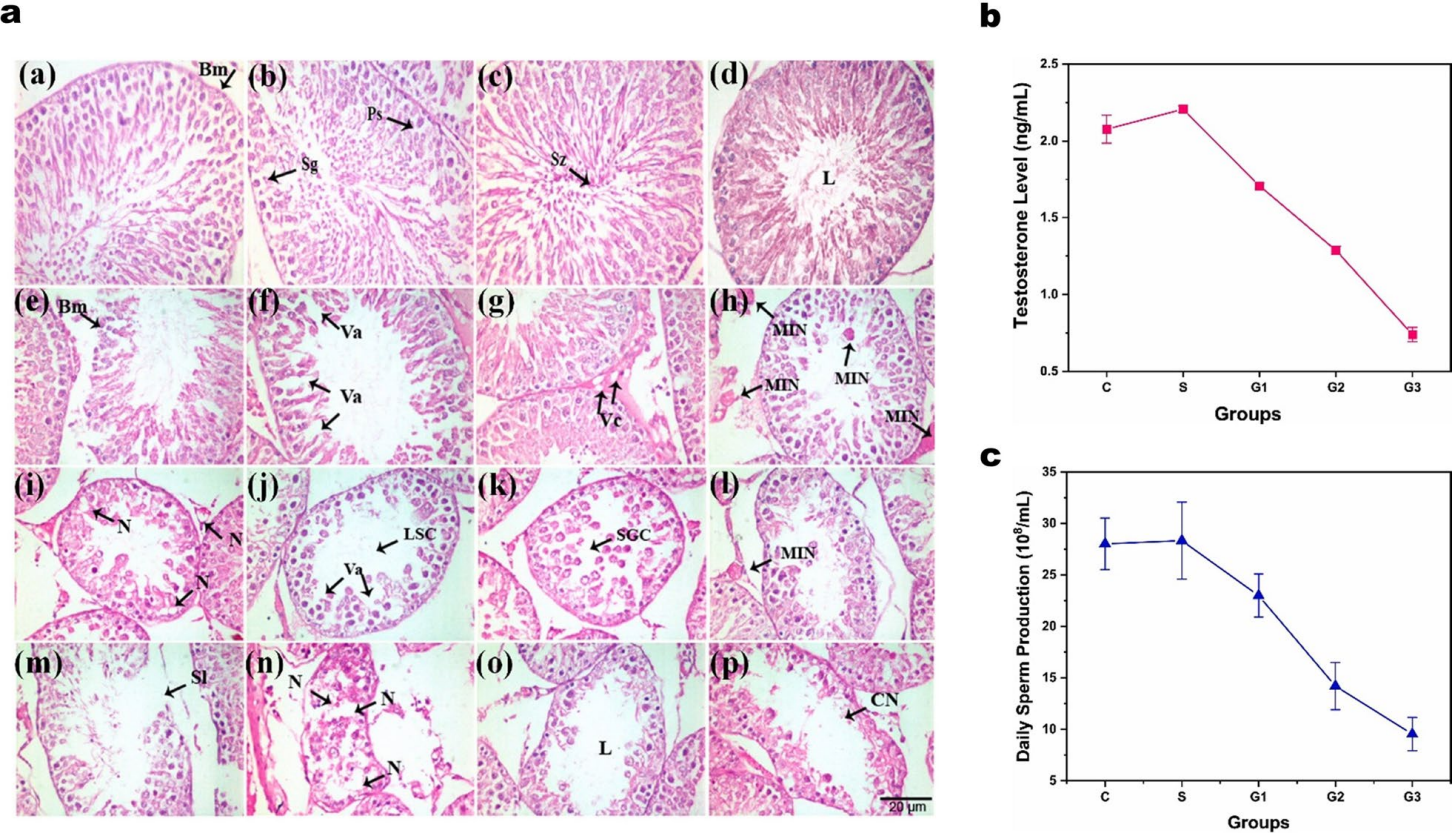
**a** Illustration of histological alterations (H&E); **a**–**d** Normal testes without Ni NPs treatment (control/placebo groups), **e**–**h** G1 (15 mg/kg BW of Ni NPs), **i**–**l** G2 (30 mg/kg BW of Ni NPs) and **m**-**p** G3 (45 mg/kg BW of Ni NPs). **b**, **c** Testosterone level and the sperm production of male Sprague Dawle rats in control and Ni NPs treated groups after 14 days of exposure. Reproduced with permission [74]. Copyright 2023, Elsevier

In addition to Ni NPs, there have also been some reports on the toxic effects of NiO NPs on the reproductive system.Iftikhar et al. conducted a study to assess the male reproductive toxicity of Ni NPs measuring 56 nm in Sprague Dawley rats [74]. Healthy rats weighing between 200 and 220 g were chosen for the toxicity evaluation and were divided into five groups: a negative control group (0 Ni NPs), a placebo group (0.9% saline), and three treated groups receiving different doses of Ni NPs (15, 30, and 45 mg/kg body weight). After 14 days of intraperitoneal exposure, the findings showed that the highest dose (45 mg/kg) resulted in a significant decrease in body weight, serum testosterone levels, and daily sperm production (Fig. 7). Conversely, the testis index, nickel accumulation, and histological alterations—including necrosis in the basement membrane and seminiferous tubules, along with vacuole formation—were observed to increase with higher doses of Ni NPs. This research establishes a foundational understanding of Ni NPs toxicity concerning the male reproductive system and may inform risk assessments for products containing Ni NPs.

#### Comparative and multisystem effects

Nanomaterials can have multisystem effects, impacting various body systems simultaneously. They can induce oxidative stress, inflammation, and cellular damage across organs, including the respiratory, cardiovascular, nervous, renal, and integumentary systems. These effects may lead to respiratory issues, cardiovascular diseases, neurotoxicity, nephrotoxicity, and skin disorders. Studies have shown that inhaled NiO NPs not only affect the pulmonary system but also cause significant damage to the liver and kidneys. Furthermore, NPs can transfer from the nasal mucosa along the olfactory tract, causing damage to corresponding structures in the brain [85]. Additionally, NBNs can simultaneously affect both the brain and the immune system. Shipelin et al. explored the acute and subchronic oral toxicity of Ni NPs in rats [147]. The researchers used two NP formulations (Ni NP1 and Ni NP2), which, according to electron microscopy data, have spherical particles with ~ 53.7 and 70.9 nm, respectively. In the acute toxicity study, a single dose of 2000 mg/kg body weight of the two types of Ni NPs was administered by gavage to 8-week-old male and female Wistar rats. In the subchronic experiment, male Wistar rats, initially aged several weeks, were subjected to a 92-day treatment with Ni NP1, Ni NP2, and a conventional soluble salt form of nickel (Ni basic carbonate). The study revealed the bioaccumulation of Ni NPs and nickel salts in the kidneys, while no significant accumulation was observed in the liver and spleen. Notably, the brain and the immune system were identified as the primary organs affected by Ni NPs in this subchronic study.

Nanomaterials can pose significant cardiopulmonary toxicity due to their small size, which allows them to penetrate deep into the lungs and enter the bloodstream. This can lead to inflammation, oxidative stress, and other adverse effects on both the respiratory and cardiovascular systems. In a study by Garces et al., mice were intranasally instilled with silicon NPs containing nickel (Ni-NP) or without nickel (NP) [148]. Lung, plasma, and heart samples were collected 1h post-exposure to assess redox metabolism. The results showed that NP primarily accumulated in the lungs, leading to a significant increase in tissue oxygen consumption. In contrast, Ni-NP generated ROS through NADPH oxidase (NOX) activity and increased the rate of mitochondrial H_2_O_2_ production. Additionally, the GSH/GSSG ratio in the lungs indicated a shift in the antioxidant system, resulting in a decreased redox state and increased SOD activity, which led to elevated phospholipid oxidation. In the circulatory system, there was a reduction in polymorphonuclear leukocyte activation and a lower GSH/GSSG ratio, with phospholipid oxidation observed in plasma samples from the Ni-NP group. Consequently, in distant organs such as the heart, Ni-NP inhalation altered the tissue redox state. The study concluded that exposure to nickel-containing silicon NPs induced redox metabolism changes, leading to cardiopulmonary toxicity. Overall, exposure to NBNs through a particular route can lead to multi-organ damage, mainly due to a combination of factors such as their high surface area and reactivity, biological distribution and transport, persistence, oxidative stress and inflammatory responses, immune system reactions, direct cytotoxicity, and genotoxicity.

## Regulatory aspects and risk assessment

### Susceptible populations

The most at-risk population for NBNs exposure is workers in industries involving the production, handling, and processing of NBNs, such as manufacturing, welding, and mining [9, 155]. These individuals may be at higher risk of exposure to airborne Ni NPs. NBNs can potentially be released into everyday life through various sources. For example, NBNs may be used in the production of consumer goods such as electronics, textiles, and personal care products. These materials could potentially release NPs during their use or disposal, leading to potential human and environmental exposure. Additionally, NBNs used in agricultural products, soil remediation, or water treatment may enter the environment and impact ecosystems, potentially leading to human exposure through food, water, or air. Individuals with pre-existing respiratory conditions, children and infants, individuals with nickel allergies or sensitivities, pregnant women and individuals with compromised immune systems, etc., may be more susceptible to the adverse effects of NBNs.

### Relevant regulations

Policies and regulations aimed at controlling the environmental and health risks of nickel involve various domains, including air quality, water quality, waste management, and occupational safety. Key regulations include the U.S. Clean Air Act, which lists nickel compounds as hazardous air pollutants and requires industries to implement Maximum Achievable Control Technology (MACT) standards [156]. The U.S. Safe Drinking Water Act provides health advisory levels for nickel in drinking water [157]. The Resource Conservation and Recovery Act (RCRA) has strict regulations for the treatment and disposal of nickel-containing waste, with nickel being classified as a component of hazardous waste. Different countries may have varying soil quality standards to limit nickel concentrations in soil, aiming to prevent adverse effects on ecosystems and human health. Occupational safety is addressed by the Occupational Safety and Health Administration (OSHA), which sets exposure limits for nickel in the workplace. For example, for insoluble nickel compounds, the current permissible exposure limit (PEL) enforced by the OSHA, as an 8-h time-weighted average, is 1.0 mg/m^3^. Moreover, the National Institute for Occupational Safety and Health (NIOSH) recommends an exposure limit (REL) for NiO of 0.015 mg/m^3^ as a time-weighted average [158]. In the European Union, the REACH regulation governs the registration and assessment of nickel and its compounds. Internationally, the World Health Organization (WHO) offers guidelines for nickel in drinking water (guideline value 0.07 mg/L), and the International Agency for Research on Cancer (IARC) classifies certain nickel compounds as carcinogenic, providing a basis for regulatory measures [159]. These regulations collectively aim to mitigate the risks associated with nickel exposure to protect both human health and the environment.

### Minimizing exposure

Environmental exposure can be minimized by reducing the release of NBNs into the workplace environment [160]. Personal protective equipment, including respirators and protective clothing, should be provided to workers to reduce exposure [161]. Workers can be trained to deal with the safe handling, storage, and disposal of NBNs to minimize the risk of exposure. This includes proper labeling, containment, and waste management practices. Regular monitoring and surveillance programs need to be implemented to assess the levels of NBNs in the workplace environment and evaluate the effectiveness of control measures.

### Mitigating toxic effects

Reducing the toxicity of nanomaterials is of great significance for their safe use [162]. There has been considerable research on strategies to mitigate the toxicity of nanomaterials. Strategies to mitigate the toxicity of nanomaterials involve several key approaches. Surface modification can enhance biocompatibility and reduce adverse interactions with biological systems. Controlled release mechanisms are designed to minimize exposure to potentially toxic ions or compounds [163]. Additionally, improving the biodegradability of nanomaterials ensures they decompose into non-toxic components, reducing long-term environmental and biological risks. These strategies collectively aim to harness the benefits of nanomaterials while minimizing their potential hazards. These strategies are of reference significance for mitigating the toxic health effects of NBNs.

Several reports have addressed the reduction of toxicity in NBNs, and we have compiled strategies in Table 3 that can serve as a reference for minimizing their toxicity. Nano-selenium can alleviate mitochondria-related apoptosis of nickel-induced in vivo and in vitro hepatotoxicity through PI3K/AKT pathway [164]. Nano-selenium has protective effect on the apoptosis of rat renal cells induced by nickel [173]. The antioxidant vitamin C shows a significant inhibitory effect on Ni NPs-induced reproductive toxicity [109]. Hesperidin (HSP) is a citrus flavonoid with powerful anti-inflammatory, antioxidant and free radical scavenging activities. Combined administration of HSP and NiO NPs significantly can improve NiO NPs-induced testicular injury and increase male fertility in rats [165]. In another study, HSP can significantly improve most hepatorenal toxicity in NiO NPs-induced male rats [129]. Additionally, epigallocatechin-3-gallate (EGCG) exhibits an inhibitory effect on the toxicity induced by Ni NPs in a mouse epidermal cell line (JB6 cells) [166]. Apigenin has also been reported to protect the liver and kidneys from oxidative damage caused by nano NiO in male rats [167]. This inhibition is likely mediated through the MAPK signaling pathway. There are also nickel chelators, such as the dietary polyphenol chlorogenic acid, which have the potential to alleviate skin allergy symptoms caused by NBNs [172].

**Table 3:** Summary of strategies that can provide a reference for reducing the toxicity of NBNs

Strategies employed	Source material of toxicity	Specific detoxification components	Reduction of toxicity effects	Refs.
Antioxidant incorporation	Ni^2+^ ions	Nano-selenium	Alleviation of mitochondria-related apoptosis in nickel-induced in vivo and in vitro hepatotoxicity	[164]
Antioxidant incorporation	Ni NPs	Vitamin C	Protective role in the reproductive system of male rats	[109]
Antioxidant incorporation	NiO NPs	Hesperidin (HSP)	Reduction of testicular injury and increase in male fertility in rats	[165]
Antioxidant incorporation	Ni NPs	Epigallocatechin-3-gallate (EGCG)	Reduction of toxicity to a mouse epidermal cell	[166]
Antioxidant incorporation	NiO NPs	Apigenin	Protection of the liver and kidneys from oxidative damage	[167]
Antioxidant incorporation	NiO NPs	Mediterranean plant P. lentiscus (PLEO)	Reduced cytotoxicity and oxidative stress in A549 cells	[168]
Coating modification	Ni NPs	Porous silica matrix	Non-hollow Ni@SiO_2_ reduced developmental toxicity in zebrafish	[169]
Coating modification	NiTi alloy	quaternized coating materials	Significant reduction of Ni^2^⁺-induced cytotoxicity through physical barrier and physical adsorption	[170]
Chelation therapy	Ni^2+^ ions	CaCO_3_ or CaPO_4_ NPs	Prevention of Ni^2^⁺ ion penetration through the skin	[171]
Chelation therapy	Ni^2+^ ions	MSN-His(6)@CGA NPs	Capture nickel to alleviate skin allergies	[172]

**Fig. 8:**
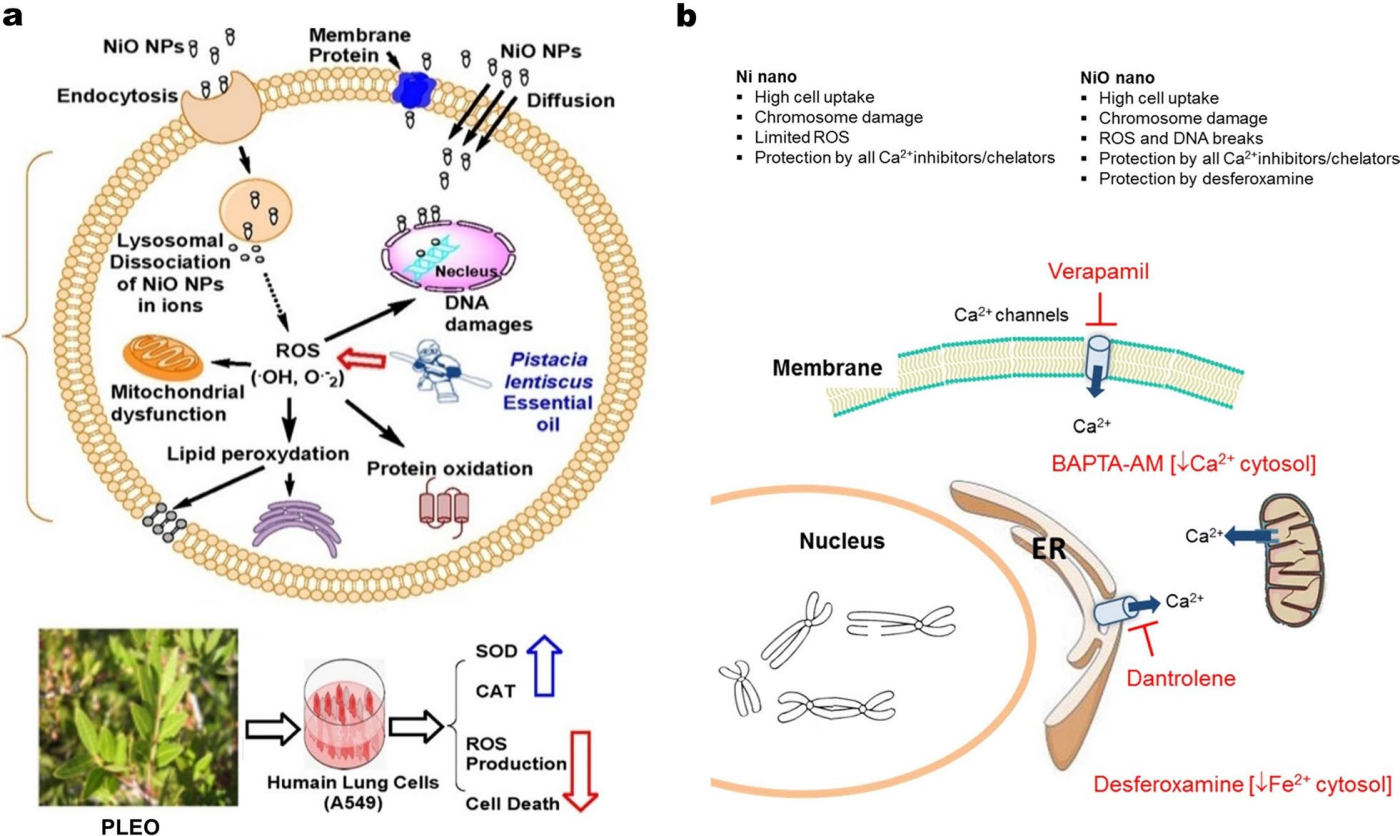
Some strategies for reducing the toxicity of NBNs. **a** NiO NPs induce cytotoxicity in A549 cells through ROS generation and impairment of the antioxidant defense system. Pistacia lentiscus essential oil provides a protective effect against this damage. Reproduced with permission. [168] Copyright 2020, Elsevier B.V. **b** Ni NPs and NiO NPs cause chromosomal damage dependent on Ca^2+^. The use of calcium inhibitors and chelators effectively prevents this chromosomal damage. Reproduced with permission [175]. Copyright 2020, Springer Nature

Today, the use of medicinal plants is the most widely used form of medicine worldwide. The use of aromatic plants as a source of interesting plant chemical substances constitutes one of the greatest scientific interests. Therefore, Khiari Mohamed and colleagues studied the essential oil of the Mediterranean plant P. lentiscus (PLEO) for its antioxidant and cell protective effects against NiO NPs-induced cytotoxicity and oxidative stress in A549 cells (Fig. 8a) [168]. The results obtained showed that NiO NPs reduced cell viability and induced oxidative stress in a dose-dependent manner, as evidenced by the induction of reactive oxygen species and a decrease in antioxidant enzyme activity. Their study also revealed that PLEO contains a high amount of pinene-4-ol (11.49%), elemol (8.64%), α-terpinene (5.97%), hinokitiol (5.19%), caryophyllene (5.10%), and δ-terpinene (4.86%). In this cellular model, PLEO demonstrated effective antioxidant capacity by improving cell viability, scavenging ROS, and enhancing the endogenous antioxidant system against NiO NPs.

Research has shown that other NPs can significantly alter the bioavailability of nickel. For example, Doria-Manzur et al. discovered that at a nickel contamination level of 5 ppm, the presence of 50 and 100 ppm ZnO NPs reduced nickel uptake by approximately 43% and 47%, respectively [174]. Additionally, the results indicated that at 50 ppm of ZnO NPs, both the phytotoxic effects of nickel and the NPs themselves were mitigated, leading to higher dry biomass yield in plants. This study highlights the potential of ZnO NPs in phytoremediation by reducing nickel uptake in gluten-free crops such as sorghum. It also provides insights into mitigating the toxicity of NBNs. Yuan et al. demonstrated that applying a thin layer of a glycerin-based moisturizer containing CaCO_3_ or CaPO_4_ NPs on isolated pig skin (in vitro) and mouse skin (in vivo) can prevent Ni^2+^ ions from penetrating the skin [171]. The NPs capture Ni^2+^ ions through cation exchange and retain them on the skin surface, which can then be removed with simple washing. The amount of NPs required to achieve the same efficacy as the chelating agent EDTA is reduced by approximately 11-fold. Incorporating NPs with sizes below 500 nm in topical creams could serve as a viable approach to reduce exposure to metal ions known to induce skin irritation.

Recently, Mahoney et al. investigated the toxicity of three structurally distinct nickel-silica nanomaterials as prototypical complex engineered nanomaterials (CENs): simple surface-deposited Ni-SiO₂, and hollow and non-hollow core–shell Ni@SiO₂ materials (with ~ 1–2 nm Ni NPs embedded into porous silica shells, with or without a central cavity, respectively) [169]. They evaluated the toxicity of these three materials using zebrafish. The results showed that exposure to Ni-SiO₂ and hollow Ni@SiO₂ led to abnormalities in zebrafish larval motor function, indicating developmental toxicity, while non-hollow Ni@SiO₂ showed no toxicity. These observations suggest that the toxicity of Ni-SiO₂ and hollow Ni@SiO₂ may partly result from increased effective exposure at the bottom of the well due to rapid settling. Overall, the data suggest that embedding nickel NPs in a porous silica matrix can mitigate their toxicity without compromising functional properties.

By analyzing the toxicity mechanisms of NBNs, insights can also be provided for proposing strategies to modulate toxicity. Bucchianico and colleagues conducted an in-depth investigation into the genotoxicity of well-characterized Ni and NiO NPs in BEAS-2B cells to discern possible mechanisms [175]. The results have shown that Ni and NiO NPs, as well as Ni ionic species, triggered chromosomal damage in a human lung cell line. The study provided evidence for a mechanism that does not necessarily require cellular uptake but depends on the modulation of intracellular calcium and iron. NiO-induced cell death in the present model was also shown to be calcium-dependent (Fig. 8b). Therefore, regulating calcium ion concentration could potentially reduce the toxicity of nickel-based materials, and it is anticipated that such detoxification efforts will be explored.

## Conclusions, challenges and future perspectives

**Fig. 9:**
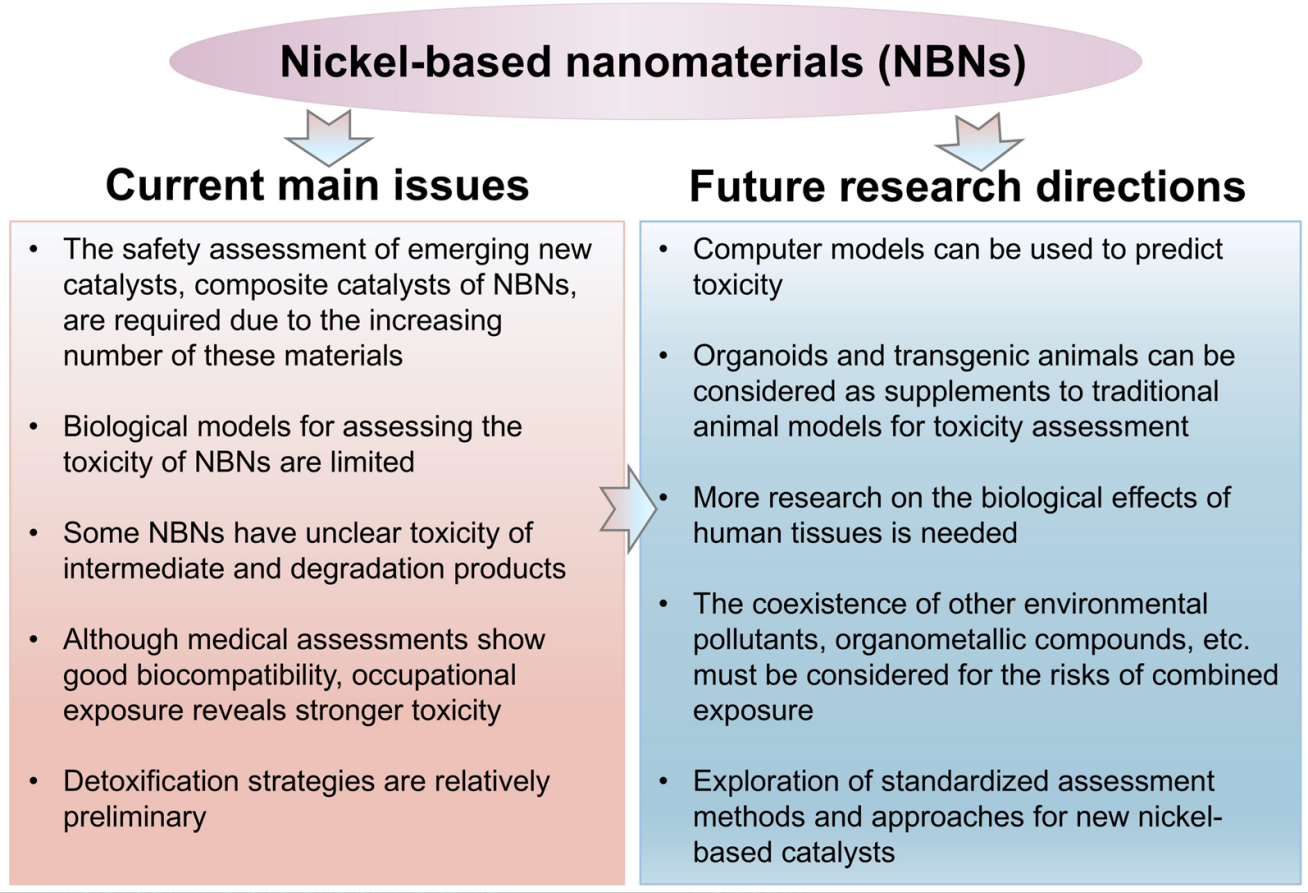
Current issues and future directions in the toxicity assessment of NBNs

In conclusion, this review provides a comprehensive overview of the toxicity of NBNs, addressing their physicochemical properties, exposure routes, health and environmental impacts, factors influencing toxicity, mechanisms of toxicity, and regulatory aspects. It underscores the importance of continued research efforts to ensure the safe and sustainable use of NBNs in various applications. The future applications of NBNs are poised to expand significantly as research and technology advance. In the energy sector, they are expected to play a crucial role in the development of more efficient and sustainable energy storage systems, such as next-generation batteries and supercapacitors. In electronics, they may contribute to the miniaturization and enhancement of devices through advanced magnetic and conductive properties. In healthcare, their potential for targeted drug delivery and improved imaging techniques could revolutionize medical diagnostics and treatments. Additionally, their use in environmental applications might grow, focusing on more effective methods for pollution control and resource recovery. As these materials continue to be engineered with greater precision, their versatility and functionality are likely to unlock new possibilities across various industries. The evaluation of the toxicity of NBNs is an essential aspect of ensuring their safe use in various applications, including electronics, catalysis, and medicine. Given the unique properties of nanomaterials that differ significantly from their bulk counterparts, specialized approaches are required to assess their potential health and environmental impacts. We have summarized the current main issues and future research directions in the study of the biological effects of NBNs in Fig. 9. Below, we will detail several future directions for the toxicity assessment of NBNs.

### Comprehensive characterization and investigation of new NBNs

Before toxicity assessments, it is crucial to thoroughly characterize the nanomaterials. This includes determining their size, shape, surface area, chemical composition, surface charge, and agglomeration state, as these factors can significantly influence their biological interactions and toxicity. However, in current research, only a few influencing factors on toxicity have been studied, and there is limited research on the impact of crystal structure of nickel-based nanomaterials on toxicity. Furthermore, current toxicity studies are only focused on several types of NBNs, such as NiO, nickel hydroxide (Ni(OH)_2_), and NiSe_2_. However, the biosafety assessments of other valuable NBNs, such as NiS, Ni_2_P, or new NBNs, also need to be explored to provide references for the development of new nickel-based nanocatalysts. For example, in biomedical applications, many novel NBNs are being developed for cancer treatment. A specific example is the nickel silicate nanoplatforms (LNS NPs), which can generate sufficient superoxide radicals under 660 nm laser irradiation, even inducing apoptosis in tumor cells in severely hypoxic environments, thereby enhancing the effectiveness of PDT for tumors [176]. However, research on health risk prevention strategies for these novel NBNs is still limited.

### Use of relevant biological models

Currently, the toxicological assessment of NBNs is relatively limited. With the advancement of science and technology, future toxicity testing should utilize relevant in vitro models (e.g., organoids and organ-on-a-chip systems) and in vivo models (e.g., transgenic animals) that can mimic exposure routes and potential targets in humans and the environment. Additionally, human stem cell models can be used by differentiating human stem cells into various cell types, such as liver cells and cardiac cells, for toxicity assessment and drug screening. The use of microbiome models can also be considered to study the interaction between microbiomes and toxins, exploring their role in toxic metabolism and detoxification. Ethical considerations, particularly in the use of animal models, should also be addressed by promoting alternative methods whenever possible.

### Advanced analytical techniques and standardization and validation

Employing advanced analytical techniques such as high-resolution microscopy, spectroscopy, and omics technologies (genomics, proteomics, and metabolomics) can provide detailed insights into the interactions between nanomaterials and biological systems. In evaluating the distribution and metabolism of NBNs within biological systems, methods such as fluorescence labeling and imaging, radioactive tracing, and MRI can be used in combination. For instance, in fluorescence labeling and imaging techniques, the photosensitive properties of naturally derived compounds with good biodegradability (such as hypocrellin) are used to label or detect the distribution and metabolism of nickel-based nanomaterials in vivo, thereby aiding in the assessment of their toxicity [177]. In addition, using artificial intelligence technology for toxicity prediction and screening can quickly identify potential toxic substances and reduce the use of experimental animals. Overall, developing standardized and validated testing protocols for NBNs is crucial for ensuring reproducibility and comparability of toxicity data. This also includes the need for reference materials and inter-laboratory comparisons.

### Interdisciplinary collaboration and regulatory

Addressing the complexities of nanotoxicology requires collaboration across disciplines, including chemistry, biology, materials science, toxicology, and environmental science. This interdisciplinary approach can enhance our understanding of nanomaterials' behavior and interactions in biological and environmental systems. Additionally, regulatory frameworks should be updated to include specific guidelines for the evaluation and safe use of NBNs. Engaging with the public and stakeholders about the risks and benefits of NBNs is essential. Transparent risk communication can help in building trust and informed decision-making regarding the use of these materials.

By implementing these strategies, researchers and regulatory bodies can better evaluate the toxicity of NBNs, ensuring their safe and responsible utilization in various applications.

## Data Availability

No datasets were generated or analysed during the current study.

## Abbreviations

A549: Human lung epithelial cells
ALF: Artificial lysosomal fluid
AM: Alveolar macrophages
AMF: Alternating magnetic field
ARDS: Adult respiratory distress syndrome
ART: Artemisinin
ATF: Transcription factor
BALF: Bronchoalveolar lavage fluid
BAs: Bile acids
BEAS-2B: Human lung epithelial cells
Caco-2: Human intestinal epithelial cells
CCL2: Monocyte chemoattractant protein-1
CDT: Chemodynamic therapy
CENs: Complex engineered nanomaterials
CNS: Central nervous system
DCF: Dichlorofluorescein
DDR: DNA damage response
DFO: Deferoxamine
Drp1: Dynamin-related protein 1
EGCG: Epigallocatechin-3-gallate
eIF-2α: Eukaryotic initiation factor-2α
EMT: Epithelial–mesenchymal transition
ER: Endoplasmic reticulum
GSH-Px: Glutathione peroxidase
Hb: Hemoglobin
HDF: Human dermal fibroblasts
HepG2: Human hepatocellular carcinomas
HIF-1α: Hypoxia-inducible factor 1α
HSP: Hesperidin
IL-8: Interleukin-8
IRE-1: Inositol-requiring enzyme 1
LDH: Lactate dehydrogenase
lncRNA: Long noncoding RNA
LPS: Lipopolysaccharide
MAPK: Mitogen-activated protein kinase
MCP-1: Monocyte chemoattractant protein-1
MDA: Malondialdehyde
MEG3: Maternally expressed gene 3
MIP-1α: Macrophage inflammatory protein-1α
miR-21: MicroRNA-21
MMPs: Matrix metalloproteinases
mNiO: Mesoporous nickel oxide
mNiO–Tb: Mesoporous nickel oxide terbium
MNPVs: Magnetic nanoparticle-vesicle assemblies
MPs: Microparticles
MSM: Malignant skin melanoma
NAC: N-acetylcysteine
NBNs: Nickel-based nanomaterials
Ni FPs: Nickel fine particles
NiO: Nickel oxide
NIR: Near-infrared
NO: Nitric oxide
NPs: Nanoparticles
PDA: Polydopamine
PEG: Polyethylene glycol
p-eIF-2α: Phosphorylated eIF-2α
PERK: Pancreatic ER kinase
Pink1: PTEN-induced putative kinase 1
PT: Proximal tubular
PTT: Photothermal therapy
RCD: Apoptosis
ROS: Reactive oxygen species
SD: Sprague–Dawley
TEM: Transmission electron microscopy
TGF-β1: Transforming growth factor-β1
TIMPs: Tissue inhibitors of metalloproteinases
TNF-α: Tumor necrosis factor-alpha
UPR: Unfolded protein response
UTI: Urinary tract infection
